# Hepatitis B virus is a stealth virus that minimizes proteomic and secretomic changes in primary human hepatocytes

**DOI:** 10.1099/jgv.0.002170

**Published:** 2025-11-07

**Authors:** Karolína Štaflová, Kamila Clarová, Michal Doležal, Martin Hubálek, Alena Křenková, Jan Hodek, Iva Pichová, Aleš Zábranský

**Affiliations:** 1 Institute of Organic Chemistry and Biochemistry of the Czech Academy of Sciences, Prague, Czech Republic

**Keywords:** bulevirtide, hepatitis B virus, host factor, proteomics, reticulocalbin-2, secretome, virus–host interaction

## Abstract

Hepatitis B virus (HBV) is a hepatotropic DNA virus that infects over 250 million people worldwide and causes serious liver diseases. HBV infection can modulate host cellular processes, potentially inducing proteomic changes in hepatocytes. In this study, we investigated how acute HBV infection alters the proteome and secretome of primary human hepatocytes, a physiologically relevant *in vitro* model that retains essential liver-specific functions. Protein-level changes in cell lysates and culture supernatants were quantified 8 days post-infection using data-independent acquisition MS. We used HBV infection in the presence of the entry inhibitor bulevirtide as a control to separate the effects of productive infection from those caused by inoculum-associated components. Despite robust infection, active HBV replication induced only subtle changes in host protein levels. Orthogonal validation of MS-identified candidates confirmed reticulocalbin-2 as a novel host factor downregulated during productive HBV infection. The functional role of candidate proteins identified by MS was assessed *in vitro* by siRNA-mediated knockdown and measurement of viral replication markers. Knockdown had no impact on viral RNA or antigen levels, suggesting that the observed proteomic changes may reflect stress responses or broader modulation of the hepatic microenvironment. Our findings support the concept of HBV as a stealth virus and underscore the importance of carefully controlled experimental systems for studying host responses to infection *in vitro*.

## Data Summary

Supplementary material for this article is available via Figshare: https://doi.org/10.6084/m9.figshare.30247720.v1 [1]. The MS proteomics data are available via ProteomeXchange with identifier PXD053792, token: CcU5Ple2687t.

## Introduction

Hepatitis B virus (HBV) is an enveloped DNA virus from the *Hepadnaviridae* family that infects human hepatocytes. HBV causes acute and chronic infections, with the chronic HBV infection representing a major global health burden because of its potential to cause severe liver diseases, including cirrhosis and hepatocellular carcinoma (HCC). Although current antiviral therapies can suppress viral replication and reduce the risk of disease progression, they only rarely achieve a cure [2].

Advancing our understanding of HBV pathogenesis and developing more effective therapies require detailed investigation of virus–host interactions. HBV infection modulates cellular pathways essential for viral replication and persistence [3,4]. Because these changes can manifest at the protein level, MS-based proteomics is a powerful approach to identify HBV-driven alterations in host cells.

To date, most proteomic studies in the HBV field have focused on patient-derived liver tissue or serum samples [5,10]. These studies provide valuable insights into disease mechanisms and have helped to identify potential biomarkers for diagnosis and disease monitoring. However, such samples represent a highly complex *in vivo* environment, including the effects of immune responses, inflammation, fibrosis and other pathological processes, making it difficult to identify the direct impact of HBV infection.

To overcome these limitations, cell culture models of HBV infection provide a more controlled and defined setting for identifying virus-specific proteomic changes. Several studies have used liver cancer-derived cell lines transfected with recombinant HBV DNA to characterize host responses [11,13]. Others have used stably transfected HepG2-derived cell lines, comparing HBV-expressing cells to the parental cells [14,18]. While these systems are useful for identifying HBV-related cellular pathways, they do not fully replicate the natural infection. Transfection bypasses early steps of the viral life cycle, and tumour-derived cell lines often carry oncogenic mutations and altered signalling pathways that may distort their response to infection [19,20].

Among *in vitro* models, primary human hepatocytes (PHHs) represent the most physiologically relevant system, as they retain essential liver-specific functions and a functional innate immune response. This makes them the preferred model for studying HBV biology and virus–host interactions *in vitro* [21,22].

Two recent studies investigated the effects of HBV infection on the proteome and phosphoproteome of PHH, offering valuable insights into how HBV may modulate host pathways [20,23]. However, both studies used polyethylene glycol (PEG)-precipitated supernatants from HBV-producing cell lines as the viral inoculum. As a result, their findings may have been influenced by non-infectious components co-precipitated with the virus, making it difficult to distinguish changes driven by productive HBV infection. To overcome this limitation, we included HBV infection in the presence of the entry inhibitor bulevirtide (BLV), to distinguish replication-dependent effects from those caused by inoculum-associated components.

In this study, we used label-free quantitative proteomics to investigate protein-level changes induced by *in vitro* HBV infection in PHH and their corresponding culture fluids. To our knowledge, this is the first study to address replication-dependent proteomic alterations in PHH while accounting for inoculum-induced effects, by including BLV, an entry inhibitor that blocks HBV binding to its receptor, as a control. Our findings demonstrate that the majority of proteomic alterations observed following HBV inoculation are attributable to non-infectious components present in the viral stock. These results reinforce the concept of HBV as a ‘stealth virus’, which induces only subtle changes in host cells during productive infection.

## Methods

### Cells

PHHs [#M00995-P, lot SRT (donor 1) or lot EFW (donor 2), BioIVT] were maintained in Williams’ E medium supplemented with Primary Hepatocyte Maintenance Supplements (#CM4000, Gibco), 2% FBS and 1.4% DMSO, with medium changes every 3 days.

### HBV production and purification

HBV particles were produced from HepG2.2.15 cells cultured in Dulbecco’s Modified Eagle Medium supplemented with 10% FBS and 2% DMSO. HepG2.2.15 is a stable cell line derived from HepG2 that contains two head-to-tail dimers of the HBV genome (serotype ayw, genotype D, GenBank accession number: U95551.1). Cell culture supernatants were first clarified by filtration through a 0.45 µm filter. HBV particles were then precipitated by overnight incubation in 6% PEG 8000 at 4 °C. The precipitated material was pelleted by centrifugation at 14,000 rcf for 90 min at 4 °C and resuspended in PBS supplemented with 10% FBS. Viral genome equivalents were quantified by digital droplet PCR (ddPCR) using primers and probe specific for HBV DNA (HBV DNA-F, HBV DNA-R and HBV DNA-probe; Table S1, available in the online Supplementary Material).

### HBV infection

For liquid chromatography (LC)-MS/MS analysis, PHH were seeded at 300,000 cells per well in collagen-coated 24-well plates and infected the following day. Cells were infected at a multiplicity of infection (MOI) of 1,000 viral genome equivalents (VGEs) per cell in the presence of 4% PEG 8000. After 16 h of incubation, cells were washed three times with PBS and maintained in Williams’ E medium supplemented with Primary Hepatocyte Maintenance Supplements (#CM4000; Gibco), 2% FBS and 1.4% DMSO. The HBV+BLV group was pretreated with 1 µM BLV (#P1105, Selleckchem) for 4 h before infection and then incubated with HBV in the continued presence of BLV for 16 h. The BLV-only control group received 1 µM BLV for a total of 20 h (4 h pretreatment followed by 16 h in medium containing 4% PEG 8000). The untreated control group was incubated for 16 h in medium containing 4% PEG 8000 without virus or inhibitor. All groups were cultured in the presence of 1.4% DMSO throughout the experiment. All conditions were performed in quadruplicates. Cells were harvested 8 days post-infection (dpi).

For RNA interference experiments, PHH were seeded at 150,000 cells per well in collagen-coated 48-well plates or at 50,000 cells per well in collagen-coated 96-well plates and incubated overnight. Cells were transfected with siRNA the following day, and HBV infection was performed 24 h post-transfection at an MOI of 500 VGE/cell following the same infection protocol as above.

### Immunofluorescence analysis

Immunofluorescence analysis (IFA) of HBV infection in PHH was performed as previously described [24]. Cells were fixed and stained with antibodies specific for hepatitis B core antigen (HBcAg) and hepatitis B surface antigen (HBsAg), and nuclei were counterstained with DAPI. Images were acquired using an Olympus IX81 fluorescence microscope, and infection efficiency was quantified using ScanR Analysis software (version 3.4.1, National Instruments Corporation). The primary antibodies used were rabbit monoclonal anti-HBcAg (#53, Gilead Sciences) and mouse monoclonal anti-HBsAg (#ab8636, Abcam). The corresponding secondary antibodies were Cy3-conjugated donkey anti-rabbit IgG (#711-007-003, Jackson ImmunoResearch) and Cy3-conjugated goat anti-mouse IgG (#115-165-003, Jackson ImmunoResearch).

### Quantification of secreted viral antigens

Secreted levels of hepatitis B e antigen (HBeAg) and HBsAg in cell culture supernatants were measured using either enzyme-linked immunosorbent assay (ELISA) kits (#BE103A for HBeAg, #BE101A for HBsAg, Bioneovan) or chemiluminescent immunoassay (CLIA) kits (#CL0312-2 for HBeAg, #CL0310-2 for HBsAg, Autobio Diagnostics), according to the manufacturer’s instructions.

### Cell viability assay

The CellTiter-Glo (CTG) luminescent cell viability assay, based on the quantification of ATP present in metabolically active cells, was performed according to the manufacturer’s instructions (#G7570, Promega).

### siRNA-mediated gene silencing

All transfections were performed using Lipofectamine^™^ RNAiMAX Transfection Reagent (#13778500, Invitrogen) according to the manufacturer’s instructions. Used siRNAs are listed in Table S2.

### Quantitative PCR

RNA was extracted using the Direct-zol RNA Microprep Kit (#R2062, Zymo Research) or Direct-zol-96 RNA Kit (#R2056, Zymo Research). Reverse transcription quantitative PCR (RT-qPCR) was performed using Luna Universal One-Step RT-qPCR Kit (#E3005L, New England Biolabs) on a CFX Opus 96 Real-Time PCR System (Bio-Rad). The GAPDH gene was used as a housekeeping control for normalization. DNA was isolated using the NucleoSpin Tissue kit (#740952, Macherey-Nagel), and ddPCR was performed using ddPCR^™^ Supermix for Probes (#1863010, Bio-Rad). Primer sequences used for qPCR are listed in Table S1.

### Quantification of secreted APOB

Secreted apolipoprotein B (APOB) levels in culture supernatants were measured using an ELISA kit (#KE00158-96T, Proteintech). Samples were diluted 1 : 200 and processed according to the manufacturer’s instructions.

### Western blot

Cell lysates were prepared using CelLytic M buffer (#C2978, Sigma-Aldrich), and protein concentrations were determined by BCA assay (#71285, Novagen). Equal amounts of protein (15 µg) were resolved by SDS-PAGE on 4–15% Mini-PROTEAN TGX precast gels (#4561085, Bio-Rad) and transferred to polyvinylidene fluoride membranes. Proteins of interest were detected with specific primary antibodies followed by HRP-conjugated secondary antibodies. Equal protein loading was confirmed by detecting vinculin on the same membrane. The following antibodies were used: anti-RCN2 (reticulocalbin-2; #10193-2-AP, Proteintech), anti-XPNPEP3 (Xaa-Pro aminopeptidase 3; #MA5-25639, Invitrogen), anti-ALDH4A1 (delta-1-pyrroline-5-carboxylate dehydrogenase; #MA5-56491, Invitrogen), anti-ENO3 (beta-enolase; #16421-1-AP, Proteintech), anti-vinculin (#V9131, Sigma-Aldrich), HRP-conjugated anti-mouse (#A4416, Sigma-Aldrich) and HRP-conjugated anti-rabbit (#AP307P, Sigma-Aldrich). Uncropped membrane images are provided in the supplementary material.

### MS sample preparation

Hepatocytes were scraped off the plate in PBS, pelleted at 1,000 rcf for 3 min at 22 °C and stored at −80 °C. Cell pellets were lysed in 200 µl of EasyPep buffer (#A45735, Thermo Fisher Scientific), and nucleic acids were digested using 1 µl of Pierce Universal Nuclease (#88700, Thermo Fisher Scientific). Protein content in the lysates was quantified using BCA assay, with 50 µg of each sample selected for further processing.

Cell culture fluids were collected from PHH at the time of harvesting and precleared using the Pierce albumin depletion kit (#85160, Thermo Fisher Scientific) modified for the removal of BSA according to the manufacturer’s instructions. Proteins for MS analysis were precipitated with four volumes of acetone for 16 h at −80 °C. Precipitates were centrifuged at 15,000 rcf for 10 min at 4 °C, and pellets were resuspended in EasyPep lysis buffer.

### LC-MS/MS data acquisition

Proteins were digested with 0.1 µg of trypsin in 50 mM ammonium bicarbonate at 37 °C for 16 h. The resulting peptides were separated on an UltiMate 3000 RSLCnano system coupled to an Orbitrap Fusion Lumos mass spectrometer (Thermo Fisher Scientific) as described in detail previously [25]. In data-independent acquisition (DIA) mode, the instrument acquired HCD fragmentation spectra for ions within an m/z range of 400 to 1,000 using variable isolation windows (delta m/z=16 was applied in the mass range 400–500, delta m/z=8 was applied in the mass range 500–650 and delta m/z=16 was applied in the mass range 650–1,000).

### Protein identification and quantification

DIA-NN (version 1.8.1) was used for DIA analysis in library-free mode [26]. All settings were set as default, and match between runs was enabled. The databases of proteomes from Homo sapiens (downloaded from Uniprot on the eighth of December 2020) and HBV (downloaded from Uniprot on the seventeenth of August 2021) were used for peptide and protein identification. The MS proteomics data were deposited to the ProteomeXchange Consortium via the PRIDE [27] partner repository with the dataset identifier PXD053792.

To differentiate proteins influenced by HBV infection, LC-MS/MS datasets were analysed using R (version 4.3.1). Label-free quantification (LFQ) intensities of the identified proteins were normalized using locally estimated scatterplot smoothing normalization. For missing values in the proteomic dataset, the MICE imputation method [28] was applied to facilitate downstream analysis by addressing data incompleteness. For differential expression analysis, linear models were fitted using the limma framework; statistical testing was performed with DEqMS [29], which computes peptide-count–adjusted empirical-Bayes moderated t-statistics. Resulting *P* values were corrected for multiple testing using the Benjamini–Hochberg false discovery rate (FDR) procedure. The significance thresholds of −log10 (adjusted *P* value) ≥1.3 (*P* value~0.05) and the absolute value of log2 fold change (FC) ≥0.5 were used to identify proteins with differential expression.

The dimensionality reduction technique principal component analysis (PCA) was used to evaluate the quality and consistency of the proteomic data obtained from LC-MS/MS. PCA was performed in R (version 4.3.1) using standard base functions.

### Gene set enrichment analysis

Gene set enrichment analysis (GSEA) of Gene Ontology terms in the Cellular Component category was conducted in R using the ClusterProfiler package (version 4.8.3) [30]. Proteins were ranked by intensity values, and enrichment scores for predefined gene sets were calculated. Normalized enrichment scores were obtained to allow comparison across gene sets. Statistical significance was assessed using permutation-based testing implemented in the GSEA algorithm, and *P* values were adjusted for multiple testing using the Benjamini–Hochberg FDR procedure.

### Statistical analysis

Statistical significance between siRNA-treated groups and the control was determined using one-way ANOVA followed by Dunnett’s multiple comparisons test. Analyses were performed using GraphPad Prism (version 10.4.0). A *P* value below 0.05 was considered statistically significant (*P<0.05, **P<0.01 and ***P<0.001).

## Results

### Proteomic characterization of HBV infection in PHHs

We used an *in vitro* infection model to investigate the effects of HBV infection on host cell protein expression. The infectious HBV inoculum was prepared by ultrafiltration of culture supernatants from HBV-producing HepG2.2.15 cells, followed by PEG-mediated virion precipitation [31]. This commonly used method enriches viral particles but does not yield a pure virus preparation. To assess the protein composition of the HBV inoculum, we performed qualitative LC-MS/MS analysis which identified 1,840 proteins (Table S3). GSEA of these proteins revealed enrichment of pathways related to coagulation, immune responses and extracellular matrix interactions (Fig. S1).

**Fig. 1.**
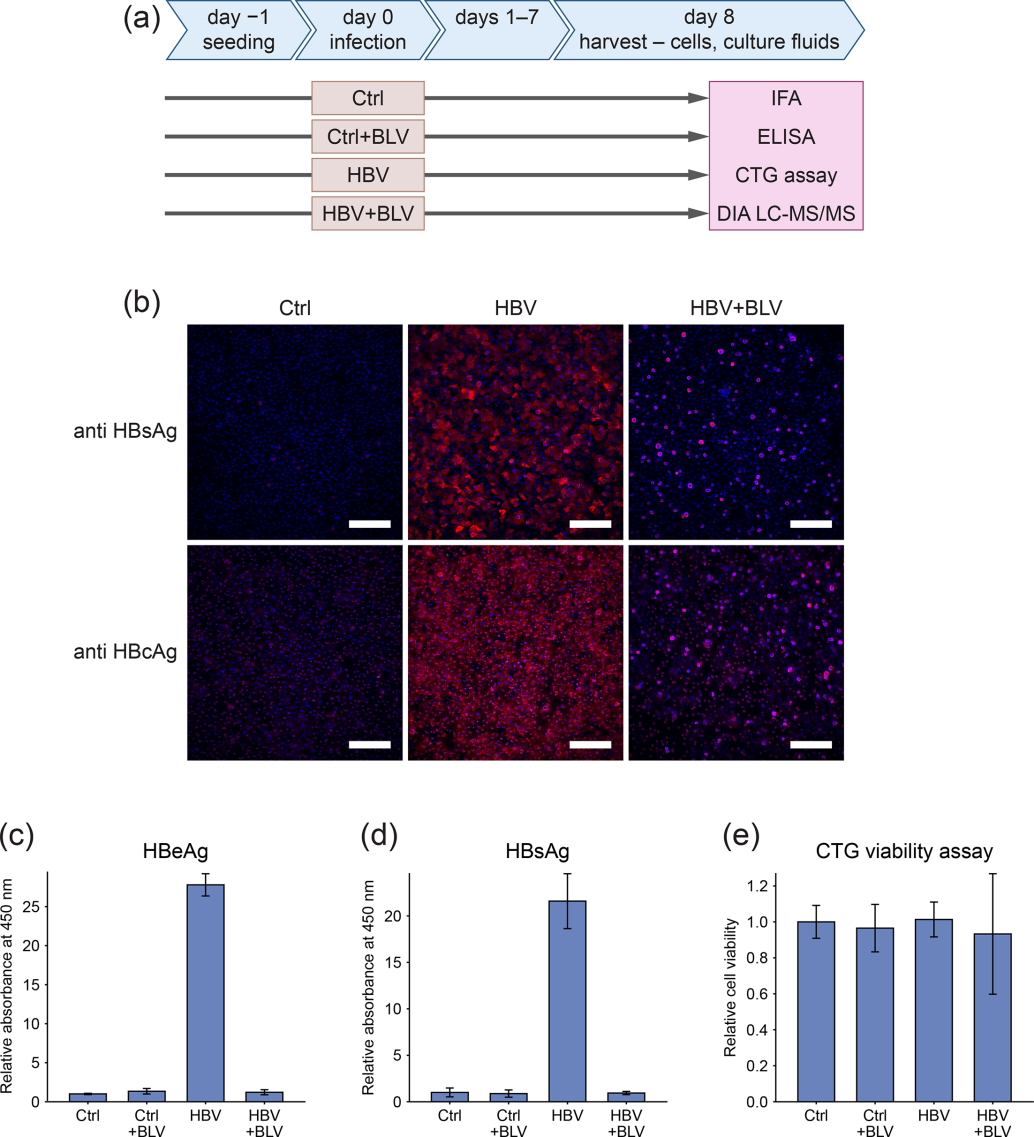
Monitoring of HBV infection in PHH. (**a**) Experimental timeline. PHHs were harvested at 8 dpi for downstream analysis. Experimental groups included uninfected control (Ctrl), BLV-treated cells (Ctrl+BLV), HBV-infected cells (HBV) and cells infected with HBV in the presence of BLV (HBV+BLV). (**b**) HBV infection was assessed by IFA of intracellular HBsAg and HBcAg. Scale bar represents 300 µm. (**c, d**) The secretion levels of (**c**) HBeAg and (**d**) HBsAg in cell culture supernatants were determined using ELISA and are presented relative to Ctrl. Data represent mean±sd. (**e**) The effect of HBV infection and BLV treatment on cell viability was determined using the CTG assay. Results are shown relative to Ctrl. Data represent mean±sd.

PHHs were infected with the HBV inoculum at an MOI of 1,000 VGE/cell in 4 replicates. To distinguish HBV-specific effects from those caused by co-purified host factors or nonproductive viral uptake, we included a control group treated with the selective HBV entry inhibitor BLV. Cells in the HBV+BLV group were pretreated with 1 µM BLV for 4 h and subsequently infected in the continued presence of the inhibitor for 16 h. Additional control groups included untreated PHH (Ctrl) and PHH treated with BLV alone (Ctrl+BLV). Eight days post-infection, cells and supernatants were collected for further analysis (see Fig. 1a for the experimental outline).

To assess infection efficiency, we performed immunofluorescence staining for HBcAg and HBsAg. HBV-infected PHH displayed a high infection rate, with ~80% of cells positive for HBcAg. In comparison, HBV+BLV-treated cells showed only ~15% positivity, and uninfected controls had no detectable signal (Fig. 1b). Secreted levels of HBeAg and HBsAg were measured by ELISA (Fig. 1c, d), confirming productive replication in HBV-infected cells and effective inhibition in the HBV+BLV group, which exhibited only background level of antigen secretion. These findings indicate that the small proportion of HBcAg- and HBsAg-positive cells observed in the HBV+BLV group likely results from nonproductive internalization of virions that failed to establish replication. Cell viability, assessed by the CTG assay, was not affected by HBV infection or BLV treatment in any condition (Fig. 1e).

For proteomic analysis, samples were subjected to LC-MS/MS using a DIA strategy. This analysis identified 5,963 proteins in cell lysates and 950 proteins in culture supernatants. The processed LC-MS/MS datasets showed good consistency among biological replicates, as reflected by condition-specific clustering observed in PCA (Fig. S2).

### Proteomic alterations in PHH cell lysates following HBV infection

**Fig. 2.**
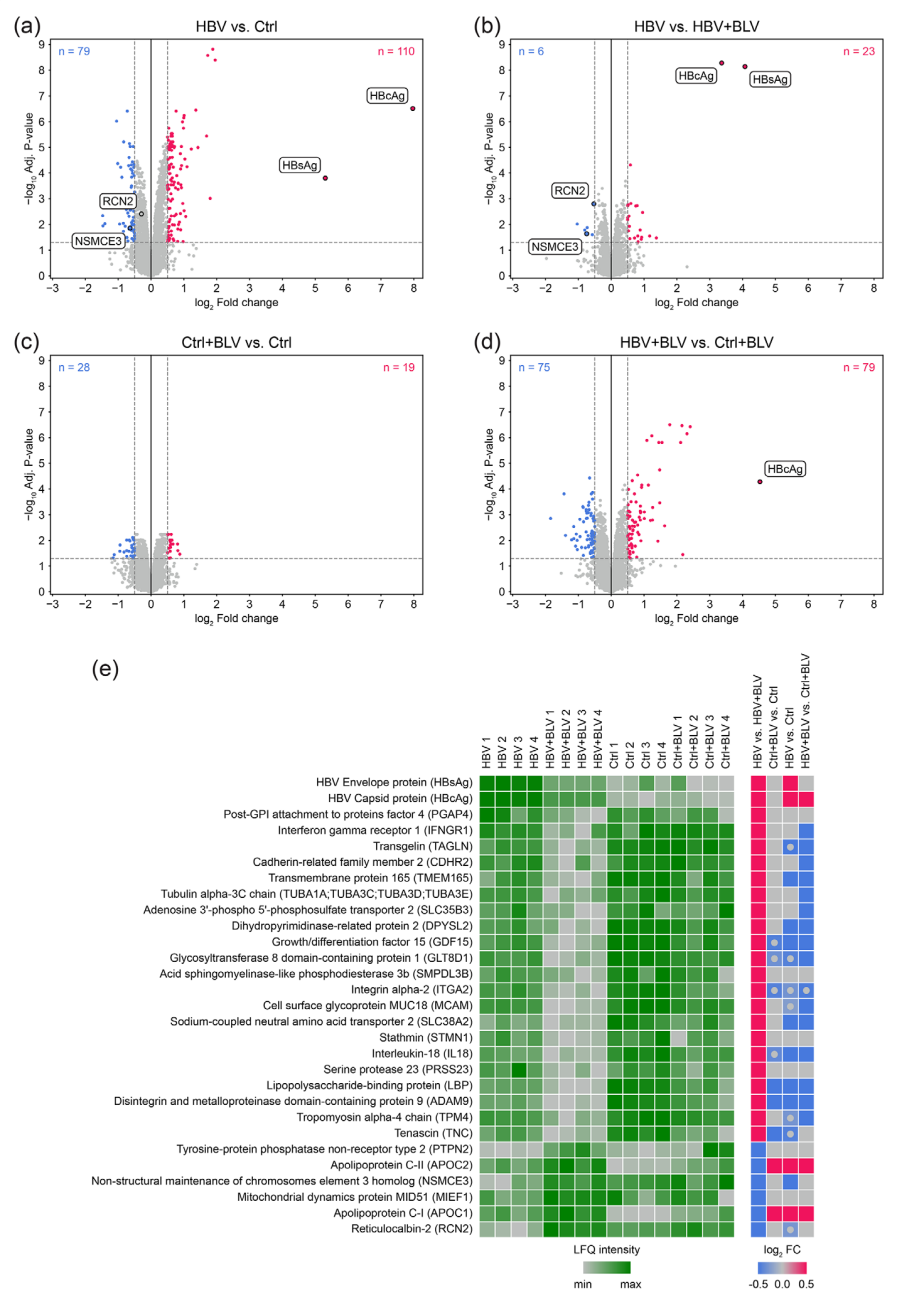
Differential analysis of proteomic changes in PHH after HBV infection. Experimental conditions included uninfected control (Ctrl), BLV-treated control (Ctrl+BLV), HBV-infected cells (HBV) and HBV-infected cells treated with BLV (HBV+BLV). (**a–d**) Volcano plots show changes in protein abundance in PHH lysates across four pairwise comparisons: (**a**) HBV vs. Ctrl, (**b**) HBV vs. HBV+BLV, (**c**) Ctrl+BLV vs. Ctrl and (**d**) HBV+BLV vs. Ctrl+BLV. Significantly upregulated proteins (log₂ FC≥0.5, adjusted *P* value≤0.05) are shown in red, and downregulated proteins (log₂ FC≤−0.5, adjusted *P* value≤0.05) are shown in blue. Statistical testing was performed using DEqMS, which applies peptide-count–adjusted empirical-Bayes moderated t-tests based on limma, followed by Benjamini–Hochberg FDR correction. Selected viral proteins (HBsAg and HBcAg) and host proteins discussed in the text are labelled. (**e**) Heatmap showing LFQ intensities of proteins differentially abundant in the HBV vs. HBV+BLV comparison. Each column represents an individual sample, and each row corresponds to one protein. LFQ intensities for individual proteins are colour-coded from minimum (light grey) to maximum (dark green). The right panel summarizes pairwise differential expression analyses for the proteins shown in the heatmap. Red and blue squares represent statistically significant upregulation and downregulation, respectively (adjusted *P* value≤0.05, |log₂ FC|≥0.5). Grey squares indicate no significant change. Squares with a grey circle mark proteins with statistically significant differences (adjusted *P* value≤0.05) that did not meet the fold-change cutoff.

To assess proteomic changes in HBV-infected PHH, we performed pairwise differential analyses of protein abundance in cell lysates across multiple conditions (Fig. 2a–d, Table S4).

Comparison between HBV-infected and uninfected PHH (HBV vs. Ctrl) revealed 110 upregulated and 79 downregulated proteins (Fig. 2a). This dataset reflects the overall changes induced by the HBV inoculum, including proteomic alterations associated with both productive and nonproductive viral internalization, as well as nonspecific effects from co-purified host factors. Among HBV-derived proteins, only HBcAg and HBsAg were detected in the lysates of infected PHH. Low-abundance viral proteins, polymerase and the regulatory protein X (HBx), were not identified.

To distinguish proteomic changes specifically associated with active HBV replication, we compared HBV-infected cells to BLV-treated HBV-infected cells (HBV vs. HBV+BLV, Fig. 2b). Notably, this comparison yielded only 23 upregulated and 6 downregulated proteins, suggesting that the majority of changes observed in the HBV vs. Ctrl comparisons are likely attributable to the inoculum (Fig. 2a vs. 2b). These findings underscore the importance of including a viral entry inhibition or similar control when analysing HBV-induced cellular responses *in vitro*.

However, BLV treatment itself may influence the proteome. To evaluate its independent effects, we compared BLV-treated uninfected PHH to untreated controls (Ctrl+BLV vs. Ctrl, Fig. 2c). This analysis identified 19 upregulated and 28 downregulated proteins, suggesting that 20 h BLV exposure induces detectable proteomic changes that persist for at least 8 days. Therefore, some alterations in the HBV vs. HBV+BLV comparison may reflect a combined effect of both HBV replication and BLV treatment.

Finally, the comparison of BLV-treated HBV-infected cells with BLV-treated uninfected cells (HBV+BLV vs. Ctrl+BLV, Fig. 2d) revealed changes associated with nonproductive HBV internalization and the presence of nonviral components in the inoculum. Some of the differentially abundant proteins in this comparison were also found in the inoculum proteome (Fig. S3, Table S5), raising the question of whether these changes reflect cellular responses triggered by inoculum-derived host proteins or are partly explained by residual carryover of these components.

Since our primary aim was to distinguish protein-level changes associated with productive HBV replication, we focused on the comparison between HBV-infected cells and BLV-treated HBV-infected cells (HBV vs. HBV+BLV). LFQ intensity-based analysis of differentially abundant proteins in this comparison is visualized as a heatmap in Fig. 2(e), covering all tested conditions. The right panel of the figure summarizes the direction of regulation (up or down) for each protein across all four pairwise differential analyses. It should be noted that the low background levels of HBcAg and HBsAg in uninfected controls (Ctrl or Ctrl+BLV) in the heatmap result from imputation applied during statistical processing of missing values.

Importantly, not all proteins differing between HBV and HBV+BLV were interpreted as HBV-driven. The right panel of Fig. 2(e) highlights that many of these proteins also changed in other pairwise comparisons, indicating potential influence from confounding factors. Among the 29 proteins in the heatmap, several showed differential abundance in the HBV vs. HBV+BLV comparison, but their regulation is likely driven by BLV treatment rather than by HBV replication itself. For example, changes in the abundance of LBP, ADAM9, TNC, APOC2 and APOC1 were also observed in the Ctrl+BLV vs. Ctrl comparison (Table S4), indicating that these effects are more likely attributable to BLV exposure than to productive infection.

Furthermore, to consider a change as infection-relevant, we expected it to appear consistently in both the HBV vs. HBV+BLV and HBV vs. Ctrl comparisons. Proteins genuinely affected by productive HBV replication should differ from both uninfected control and from cells exposed to the HBV inoculum without establishing replication.

Only the viral proteins HBcAg and HBsAg were consistently upregulated in both the HBV vs. HBV+BLV and HBV vs. Ctrl comparisons (Table 1). For other upregulated proteins, the effect was not reproduced in the HBV vs. Ctrl comparison. This discrepancy suggests that many of the observed changes were likely influenced by inoculum-associated components or BLV treatment and that productive HBV replication alone exerts only a limited impact on the host proteome in this *in vitro* model.

**Table 1.** Consistently altered proteins during HBV infection in PHH. The table shows FC values from differential proteomic analyses of HBV-infected cells compared with HBV+BLV-treated cells and uninfected controls

	**FC** HBV vs. HBV+BLV	**FC** HBV vs. CTRL
HBV Envelope protein (HBsAg)	17×	40×
HBV Capsid protein (HBcAg)	10×	251×
Non-structural maintenance of chromosomes element 3 homologue (NSMCE3)	0.60×	0.64×
Reticulocalbin-2 (RCN2)	0.69×	0.82×

Among the downregulated proteins, we identified NSMCE3 (non-structural maintenance of chromosomes element 3 homologue), a known component of the structural maintenance of chromosomes 5/6 (Smc5/6) restriction complex, which is targeted for HBx-mediated proteasomal degradation in HBV-infected cells [32,33]. NSMCE3 was significantly downregulated in both the HBV vs. HBV+BLV and HBV vs. Ctrl comparisons, consistent with previous findings. Other components of the Smc5/6 complex were not detected among the 5,963 proteins in our dataset.

Another downregulated protein was RCN2, which showed reduced abundance in both HBV vs. HBV+BLV and HBV vs. Ctrl comparisons (Table 1). However, in the latter case, the change did not meet the FC threshold. RCN2 is a calcium-binding protein localized in the endoplasmic reticulum [34]. Unlike NSMCE3, RCN2 has not previously been linked to HBV infection, and its role in this context remains unknown.

Taken together, our analysis shows that productive HBV infection *in vitro* induces only limited changes in the protein composition of PHH lysates. The majority of observed changes in protein abundance appear to result from BLV treatment, components co-purified with the viral inoculum or nonproductive viral uptake.

### The effect of HBV infection on culture fluid composition

**Fig. 3.**
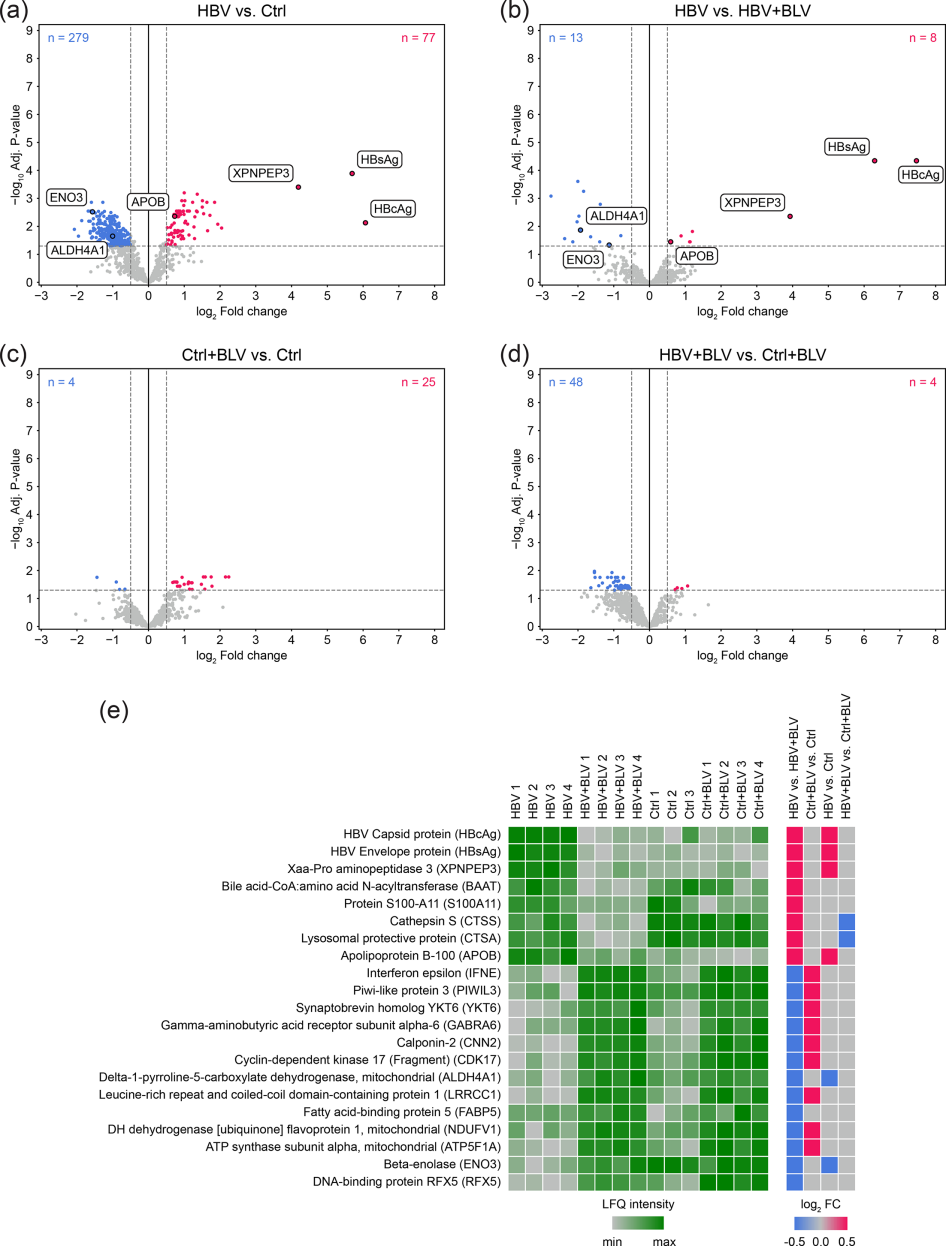
Differential analysis of proteomic changes in culture fluids from PHH after HBV infection. Experimental conditions included uninfected control (Ctrl), BLV-treated control (Ctrl+BLV), HBV-infected cells (HBV) and HBV-infected cells treated with BLV (HBV+BLV). (**a–d**) Volcano plots show changes in protein abundance in PHH cell culture supernatants across four pairwise comparisons: (**a**) HBV vs. Ctrl, (**b**) HBV vs. HBV+BLV, (**c**) Ctrl+BLV vs. Ctrl and (**d**) HBV+BLV vs. Ctrl+BLV. Significantly upregulated proteins (log₂ FC≥0.5, adjusted *P* value≤0.05) are shown in red, and downregulated proteins (log₂ FC≤−0.5, adjusted *P* value≤0.05) are shown in blue. Statistical testing was performed using DEqMS, which applies peptide-count–adjusted empirical-Bayes moderated t-tests based on limma, followed by Benjamini–Hochberg FDR correction. Selected viral proteins (HBsAg and HBcAg) and host proteins discussed in the text are labelled. (**e**) Heatmap showing LFQ intensities of proteins differentially abundant in the HBV vs. HBV+BLV comparison. Each column represents an individual sample, and each row corresponds to one protein. LFQ intensities for individual proteins are colour-coded from minimum (light grey) to maximum (dark green). The right panel summarizes pairwise differential expression analyses for the proteins shown in the heatmap. Red and blue squares represent statistically significant upregulation and downregulation, respectively (adjusted *P* value ≤0.05, |log₂ FC|≥0.5).

To extend our proteomic analysis to secreted extracellular proteins, we collected culture fluids from the last 3 days of incubation before PHH harvesting. The differential proteomic analysis of 960 identified proteins in the culture fluids is presented as pairwise comparisons (Fig. 3a–d, Table S6), following the same approach used for cell lysates.

HBV proteins HBsAg and HBcAg were detected in the culture fluids of HBV-infected cells (Fig. 3a, b), indicating the secretion of viral and subviral particles, naked capsids and HBeAg, which is indistinguishable from HBcAg by MS.

Similar to the intracellular proteomic results, the most pronounced changes in the secretome were observed when comparing HBV-infected to uninfected cultures (279 downregulated and 77 upregulated proteins; Fig. 3a), while only minor differences were detected between HBV-infected and BLV-treated HBV-infected samples (13 downregulated and 8 upregulated proteins; Fig. 3b). Both BLV treatment and non-infectious components of the inoculum affected the secretome, as shown in Fig. 3(c, d).

LFQ intensities of differentially abundant proteins in the HBV vs. HBV+BLV comparisons are presented as a heatmap across all tested conditions (Fig. 3e). Due to a technical issue, one replicate of the uninfected control medium was lost. The direction of regulation (up- or downregulation) for selected proteins across all pairwise comparisons is summarized in the right panel of Fig. 3(e).

The viral proteins HBcAg and HBsAg were again upregulated in both the HBV vs. HBV+BLV and HBV vs. Ctrl comparisons. A similar pattern was also observed for the host proteins XPNPEP3 and APOB (Table 2). XPNPEP3 is a member of the metalloprotease family [35], while APOB is a structural component of plasma lipoprotein particles.

**Table 2.** Consistently altered proteins in the secretome of PHH during HBV infection. The table shows FC values from differential proteomic analyses of culture fluids collected from HBV-infected PHH compared with HBV+BLV-treated and uninfected controls

	**FC** HBV vs. HBV+BLV	**FC** HBV vs. CTRL
HBV Capsid protein (HBcAg)	176×	67×
HBV Envelope protein (HBsAg)	79×	52×
Xaa-Pro aminopeptidase 3 (XPNPEP3)	15×	18×
Apolipoprotein B-100 (APOB)	1.51×	1.66×
Delta-1-pyrroline-5-carboxylate dehydrogenase, mitochondrial (ALDH4A1)	0.26×	0.50×
Beta-enolase (ENO3)	0.46×	0.34×

While the HBV vs. HBV+BLV analysis revealed a substantial number of downregulated proteins, the analysis of BLV-treated samples suggested that the majority of these changes could be attributed to the effects of BLV treatment (Table S6). After filtering out these BLV-associated changes, only ALDH4A1, FABP5, ENO3 and RFX5 remained downregulated, with only ALDH4A1 and ENO3 also downregulated in the HBV vs. Ctrl comparison (Table 2). ALDH4A1 is a mitochondrial enzyme involved in proline metabolism and the oxidative stress response [36]. ENO3 is beta-enolase, an enolase isoform predominantly expressed in skeletal muscle, with detectable expression also in liver tissue [37].

In summary, secretome profiling revealed that, similar to intracellular proteomic alterations, productive HBV replication induces only a limited number of changes in the extracellular proteome of PHH.

### Validation of MS-detected proteomic changes at the transcript and protein level

**Fig. 4.**
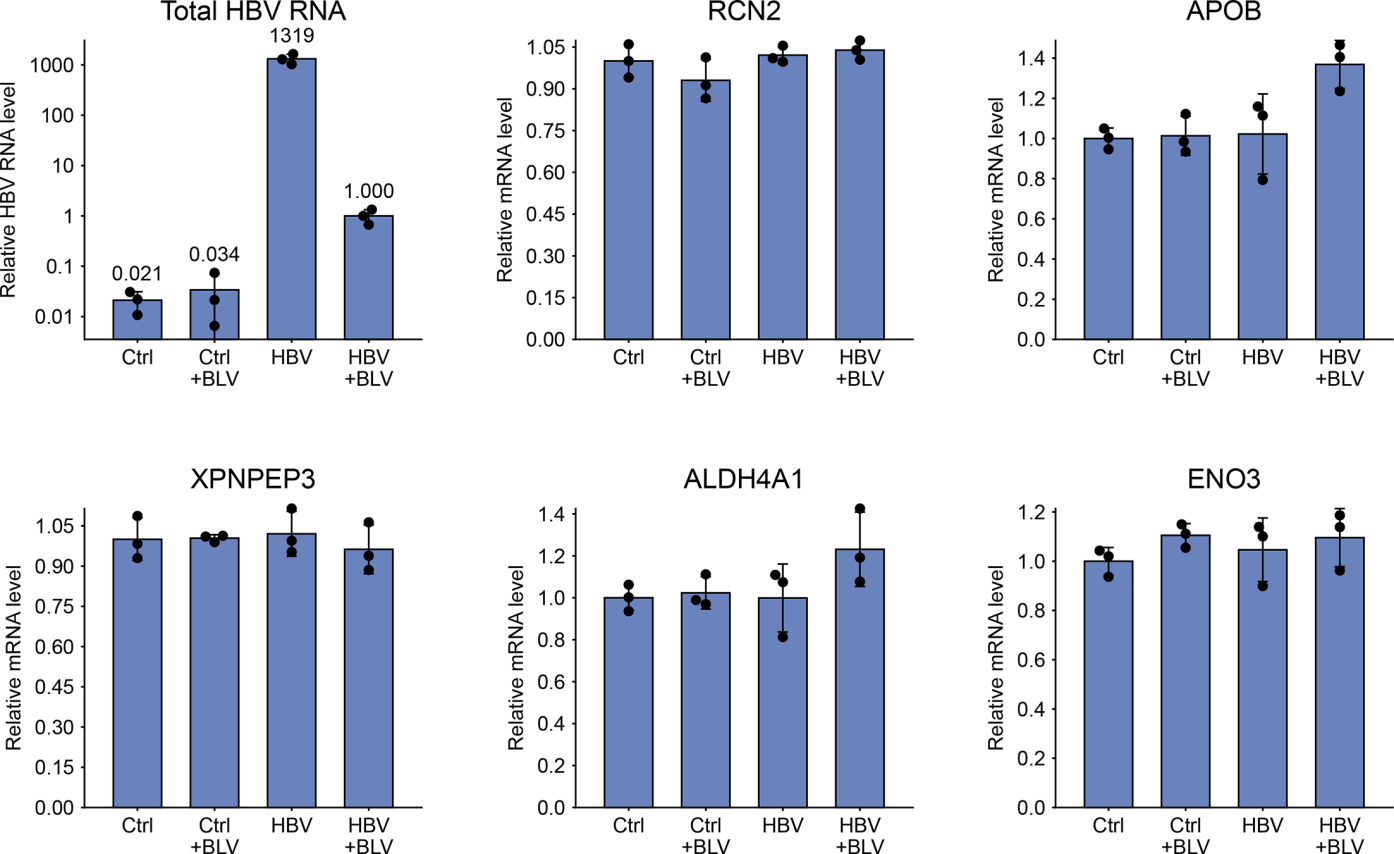
Transcript-level validation of proteomic changes by RT-qPCR. Transcript levels of RCN2, APOB, XPNPEP3, ALDH4A1 and ENO3 were analysed by RT-qPCR in PHH at 8 dpi. Gene expression was normalized to GAPDH. HBV infection was confirmed by quantifying total HBV RNA (top left), shown on a logarithmic scale and normalized to the HBV+BLV group. All other mRNA levels are shown relative to uninfected controls (Ctrl). Data represent mean±sd from a single experiment; individual points represent three replicates.

Next, we sought to validate the MS-detected proteomic changes in HBV-infected PHH at the transcript and protein level. Validation focused on the proteins reproducibly altered in both the HBV vs. HBV+BLV and HBV vs. Ctrl comparisons, namely, RCN2, APOB, XPNPEP3, ALDH4A1 and ENO3. NSMCE3 was excluded from further analysis due to its well-characterized role in HBV infection [32,33, 38]. To assess whether these changes were linked to transcriptional regulation, we performed RT-qPCR analysis of selected transcripts. PHHs from the same donor were infected under the same experimental conditions as in the proteomic assay (Fig. 1a), and total RNA was extracted for RT-qPCR. As shown in Fig. 4, the protein-level changes detected by MS were not accompanied by corresponding changes in mRNA abundance, indicating that the observed differences occur at the protein level rather than being transcriptionally regulated.

**Fig. 5.**
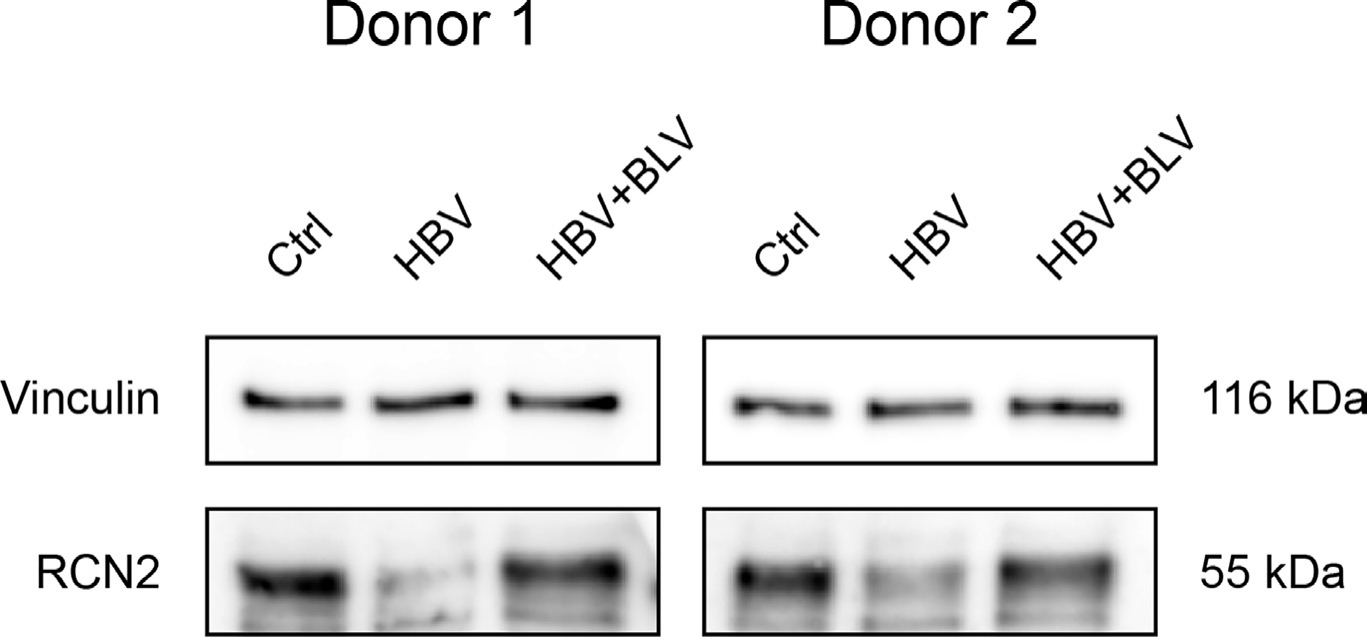
Validation of RCN2 downregulation in HBV-infected PHH. PHH from 2 donors were infected with HBV at an MOI of 1,000 VGE/cell and harvested at 8 dpi. RCN2 protein level was analysed by Western blot. A representative blot from each donor is shown. Results are representative of three replicates.

Since these changes were not reflected at the transcript level, we next examined whether they could be confirmed at the protein level by an orthogonal approach. In lysates from HBV-infected PHH, we identified two downregulated proteins, NSMCE3 and RCN2, with RCN2 downregulation not previously described in the context of HBV infection. To confirm this finding, we prepared lysates harvested at 8 dpi, matching the proteomics timeline, from the same PHH donor (donor 1) and from an additional donor (donor 2) to exclude a donor-specific effect. Productive infection was verified by measuring HBe secretion into the culture medium (Fig. S4a), and in donor 2, immunofluorescence staining for HBc indicated an infection efficiency of ~40% (Fig. S4b). Western blot analysis confirmed that RCN2 protein level decreased after HBV infection compared with uninfected controls and HBV+BLV-treated cells in both donors (Fig. 5).

**Fig. 6.**
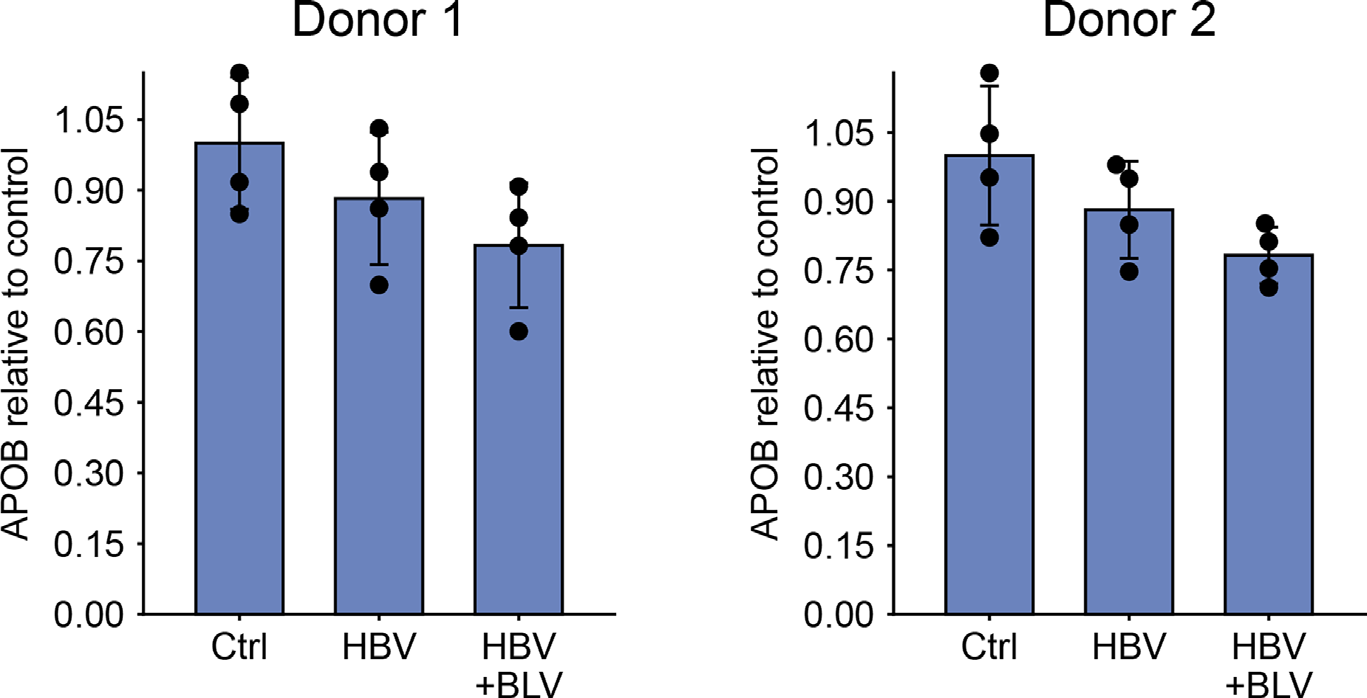
Quantification of APOB in culture supernatants of HBV-infected PHH. PHHs were infected with HBV at an MOI of 1,000 VGE/cell, and culture supernatants were collected after the final 3 days of incubation (8 dpi). APOB levels were measured by ELISA. Data represent mean±sd from a single experiment; individual points represent four replicates.

We then examined the secretome-derived candidates. Culture supernatants from the final 3 days of incubation of the same PHH samples were analysed for APOB by ELISA, but the MS-indicated differences were not confirmed (Fig. 6). The other candidates (XPNPEP3, ALDH4A1 and ENO3) were analysed by Western blot but were undetectable, even after acetone precipitation of the culture fluids. This likely reflects the higher sensitivity of MS compared with Western blot and suggests that these proteins are present in the medium at very low concentrations. As a result, these MS-based findings could not be independently validated.

Because XPNPEP3 showed the strongest MS-indicated upregulation in culture supernatants (Table 2), we further asked whether this change might also be detectable in PHH lysates. XPNPEP3 protein levels were therefore analysed by Western blot, but no increase was observed in HBV-infected PHH compared with control cells (Fig. S5).

In summary, RCN2 downregulation was confirmed to be downregulated at the protein level, establishing it as a novel host factor altered during HBV infection through a post-transcriptional mechanism. In contrast, the MS-indicated increase in APOB secretion could not be reproduced by ELISA, and the other secretome-derived candidates (XPNPEP3, ALDH4A1 and ENO3) remained below the detection limit of Western blot.

### Functional analysis of candidate host proteins in HBV infection

To assess whether consistently deregulated proteins identified by MS influence HBV replication, we performed siRNA-mediated knockdown in PHH. PHHs from donor 1 were transfected with siRNAs, infected with HBV the following day, and viral replication markers were quantified at 8 dpi.

**Fig. 7.**
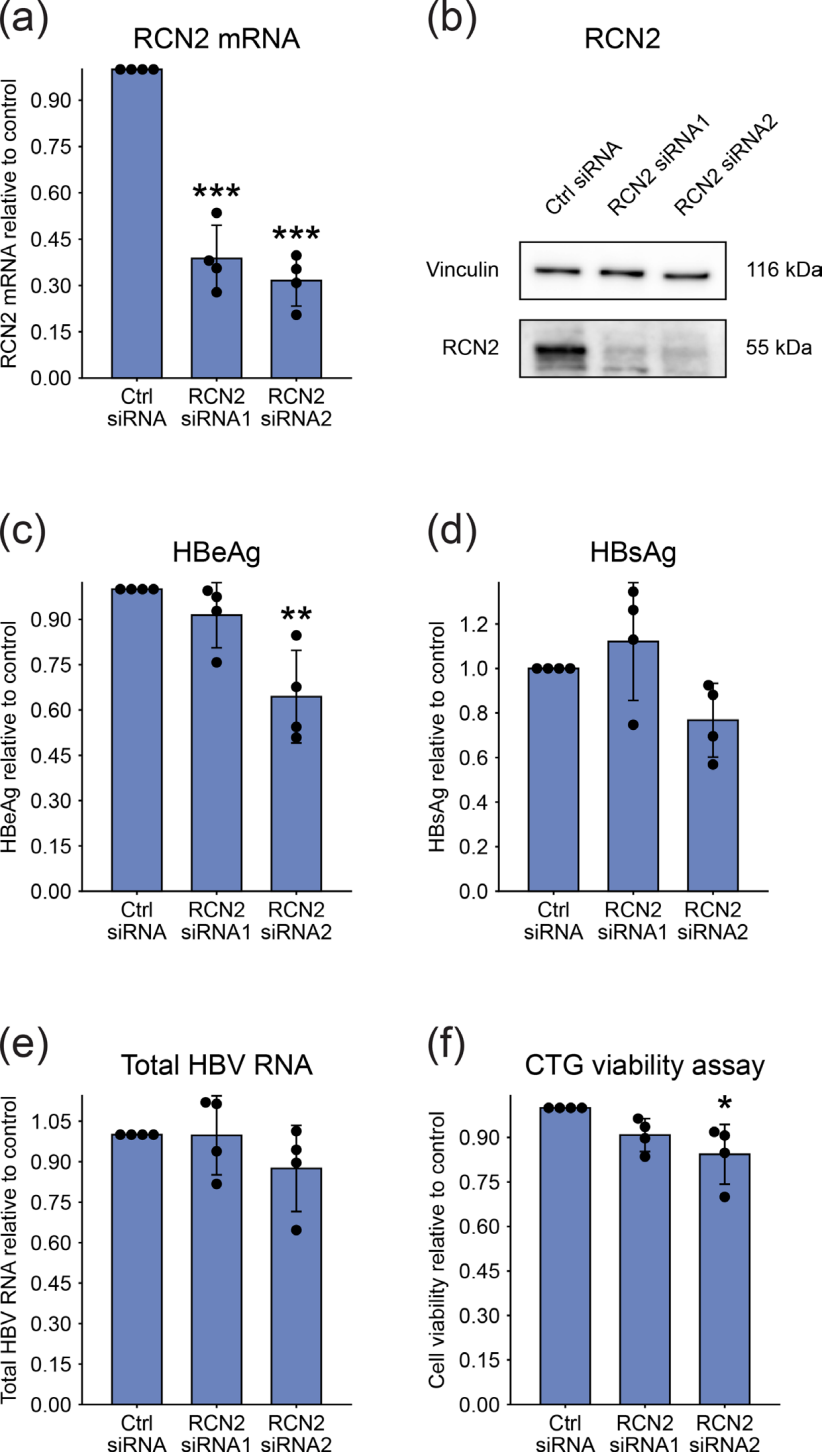
Effect of RCN2 silencing on HBV infection in PHH. (**a–e**) PHHs were transfected with siRNA and infected with HBV the following day at an MOI of 500 VGE/cell. Viral replication markers were quantified at 8 dpi. (**a**) Knockdown efficiency of RCN2 was assessed by RT-qPCR. (**b**) Knockdown of RCN2 at the protein level was confirmed by Western blot. (**c, d**) Levels of secreted HBeAg (**c**) and HBsAg (**d**) were measured in culture supernatants using CLIA. (**e**) Total intracellular HBV RNA was quantified by RT-qPCR. (**f**) Cell viability was assessed using the CTG assay 9 days after siRNA transfection. Bar plots represent mean±sd from four independent experiments. Each data point corresponds to the average of replicates within an individual experiment. Statistical significance between RCN2 siRNA groups and control was determined by one-way ANOVA (*P<0.05, **P<0.01 and ***P<0.001).

First, we analysed RCN2, which was downregulated in PHH lysates, by using two independent siRNAs to control for off-target effects. RT-qPCR confirmed that both siRNAs achieved knockdown efficiencies exceeding 60% at the end of the experiment (Fig. 7a), and this reduction was also evident at the protein level by Western blot (Fig. 7b). To determine the impact of RCN2 silencing on HBV infection, we measured secreted viral antigens by CLIA (Fig. 7c, d) and intracellular HBV RNA by RT-qPCR (Fig. 7e). Overall, we observed no consistent effect across these markers. A decrease in HBeAg secretion was observed specifically following RCN2 siRNA2 treatment, but this effect was not reproduced with siRNA1 (Fig. 7c). Moreover, RCN2 siRNA2 induced mild cytotoxicity (Fig. 7f), which may have contributed to the observed decrease in HBeAg. Together, these results suggest that RCN2 depletion does not affect HBV replication in PHH.

**Fig. 8.**
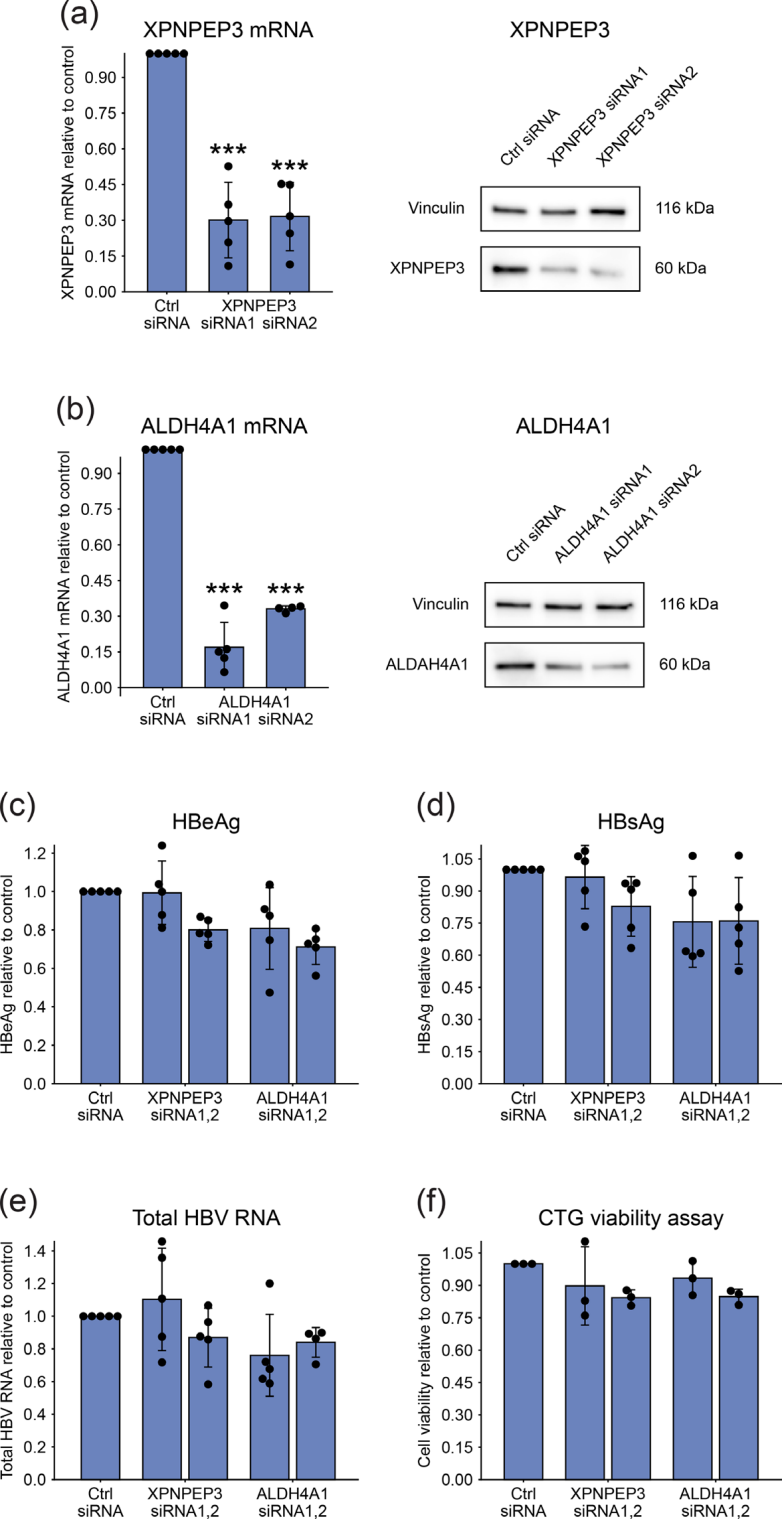
Effect of silencing secretome-associated proteins on HBV infection in PHH. (**a–e**) PHHs were transfected with siRNAs targeting XPNPEP3 or ALDH4A1 and infected with HBV the following day (MOI=500 VGE/cell). Viral replication was assessed 8 days later. (**a, b**) Knockdown efficiency was confirmed by RT-qPCR and Western blot for XPNPEP3 (**a**) and ALDH4A1 (**b**). (**c, d**) Secreted levels of HBeAg (**c**) and HBsAg (**d**) in cell culture supernatants were measured by CLIA. (**e**) Total intracellular HBV RNA was quantified by RT-qPCR. (**f**) Cell viability was assessed using the CTG assay 9 days after siRNA transfection. Bar plots represent mean±sd from at least four independent experiments, except for (**f**), which represents three independent experiments. Each data point corresponds to the average of replicates within an individual experiment. Statistical significance between siRNA-treated groups and the control was determined by one-way ANOVA (***P<0.001).

We next evaluated the secretome-associated proteins XPNPEP3 and ALDH4A1. Both genes were targeted with two independent siRNAs, and knockdown efficiency was confirmed by RT-qPCR and Western blot (Fig. 8a, b). Silencing had no significant impact on HBV replication, as assessed by secreted viral antigens (Fig. 8c, d) and intracellular HBV RNA (Fig. 8e). No substantial cytotoxicity was associated with siRNA treatment, with the maximum decrease in viability reaching ~15% (Fig. 8f).

In addition to XPNPEP3 and ALDH4A1, the MS dataset also highlighted two other consistently deregulated candidates, APOB and ENO3. APOB was excluded because the differential abundance detected by MS was not confirmed by ELISA; siRNA knockdown results are provided in Fig. S6 and, consistent with a previous study [39], showed no significant effect on HBV infection. ENO3 was excluded because efficient silencing at the protein level could not be achieved under our experimental conditions (Fig. S7).

Taken together, these results indicate that depletion of the three candidates analysed in detail (RCN2, XPNPEP3 and ALDH4A1) does not affect HBV replication in PHH. However, the secretome-associated proteins may contribute to extracellular signalling, immune evasion or modulation of the hepatic microenvironment, effects that are unlikely to be captured in our acute *in vitro* infection model.

## Discussion

Viral infections often induce changes in the host cell protein expression and secretion. In this study, we used PHHs, which retain the metabolic and immunological properties of hepatocytes *in vivo* [21,22], to investigate the impact of HBV infection on the host proteome and secretome. By including BLV, an HBV entry inhibitor, as a control, we aimed to distinguish changes associated with productive HBV replication from those caused by non-infectious components co-purified in the viral inoculum. Overall, HBV infection induced only a small number of proteomic changes, while most alterations observed following *in vitro* infection were attributable to the inoculum or to BLV treatment. Among the candidate host proteins, orthogonal validation confirmed RCN2 as a novel host factor selectively downregulated during productive HBV infection.

Previous studies investigating the host response to HBV have used a variety of models, including stably transfected hepatoma cell lines such as HepG2.2.15 or HepAD38. Comparisons between these cells and their parental cell line HepG2 have revealed hundreds of differentially expressed proteins [15,16, 18]. However, constitutive HBV expression introduces long-term adaptation and clonal selection effects, causing these lines to diverge from the parental HepG2 line [40,42]. In contrast, when HBV expression is acutely induced in HepAD38 cells in an otherwise matched background, the number of changes is considerably lower [16].

To better replicate natural infection dynamics, infection-based models using human hepatocytes have been developed. These systems support viral entry and replication through the full HBV life cycle and have been applied to study virus–host interactions using transcriptomic and proteomic approaches [20,23, 43,45]. Extending this approach, we analysed proteomic changes in HBV-infected PHH at 8 dpi using DIA MS on both cell lysates and culture supernatants. In addition to cellular proteome changes, we examined the secretome to capture extracellular alterations induced by infection, as HBV has been shown to modulate the composition of secreted and microsomal proteins [12,46].

One challenge in infection-based systems is the purity of the HBV inoculum, which can vary depending on the production method and may contain cellular contaminants [47]. For example, our proteomic analysis of the PEG-precipitated inoculum used in this study revealed over 1,800 proteins. The contamination with exosomes, microRNAs and other bioactive molecules can induce cellular responses independent of viral replication [48,49]. To address this, we used the entry inhibitor BLV, which blocks HBV binding to the sodium taurocholate co-transporting polypeptide (NTCP) receptor [50], enabling us to differentiate changes induced by viral replication from those caused by non-infectious components in the inoculum.

Using this approach, we found that HBV-infected PHH displayed 187 differentially abundant host proteins in cell lysates and 354 in culture supernatants compared to uninfected controls. However, when comparing HBV-infected cells to the HBV+BLV group, the number of differentially abundant proteins dropped markedly, to just 27 in lysates and 19 in supernatants. This suggests that the majority of observed changes were driven by components of the inoculum rather than by productive infection (Figs.2  3).

Proteomic analysis of the HBV inoculum suggests that inoculum-derived components can influence the hepatocyte proteome through several mechanisms. GSEA of the inoculum proteome (Fig. S1) showed enrichment in pathways related to coagulation, extracellular matrix and immune responses. This indicates that co-purified proteins and extracellular vesicles, concentrated during PEG precipitation, may activate signalling cascades independently of viral replication and thereby drive secondary alterations in host protein expression.

Comparison of the inoculum proteome with proteins differentially abundant in HBV+BLV vs. Ctrl+BLV samples (Fig. S3) further suggests that some inoculum proteins may persist in hepatocytes and remain detectable throughout the infection timeline. In cell lysates, this overlap was more pronounced among upregulated proteins (48 proteins) than among downregulated ones (12 proteins). The presence of these proteins at 8 dpi may be explained by the long half-lives of certain PHH proteins, which can exceed 100 h [51], allowing stable proteins to persist and potentially influence cellular pathways. However, as these values were determined for endogenously synthesized proteins, the stability of exogenously introduced proteins may differ depending on their intracellular localization.

Taken together, these findings indicate that inoculum-derived components contribute to the proteomic changes observed after HBV infection. Future studies may benefit from alternative purification strategies in place of PEG precipitation. For example, combining heparin-affinity chromatography with gradient ultracentrifugation has been reported to produce high-titre HBV stocks with reduced contamination [47].

Additionally, we show that BLV treatment alone is sufficient to induce specific and sustained proteomic changes in PHH, detectable even at 8 days post-exposure in the absence of viral infection (Figs2c  3c). Clinical studies have shown that BLV is effective and well tolerated in the treatment of chronic hepatitis delta, a satellite virus that depends on HBV for replication [52,53]. More recently, NTCP blockade by BLV has been shown to influence lipid metabolism, suggesting potential therapeutic effects beyond its antiviral activity [54,56]. Notably, several apolipoproteins involved in lipid metabolic pathways were among the proteins affected by BLV treatment in our dataset.

Some of the proteomic changes induced by BLV treatment may have influenced the comparison between HBV-infected and HBV+BLV-treated cells. For this reason, we did not consider proteins that were also differentially abundant in the BLV control as HBV-specific responses. Furthermore, to increase the likelihood of identifying biologically relevant changes, we focused on proteins that were consistently deregulated in both the HBV vs. Ctrl and HBV vs. HBV+BLV comparisons (Tables1  2). This strategy allowed us to filter out inoculum- or BLV-related effects and prioritize candidates most likely to reflect replication-dependent alterations. Only two host proteins in cell lysates and four in the secretome met these criteria, indicating that productive HBV infection triggered only subtle changes in host protein levels at 8 dpi. Consistent with previous reports describing HBV as a stealth virus [44,57, 58], we found no evidence for activation of innate immune responses.

Among the differentially abundant proteins in PHH lysates, we observed downregulation of NSMCE3, a component of the Smc5/6 restriction complex. NSMCE3 is known to undergo proteasomal degradation following HBV infection [32,33], and its downregulation shows that our infection model can capture replication-dependent host responses. Other components of the Smc5/6 complex were not identified in our dataset.

In addition to NSMCE3, we identified RCN2 as downregulated in HBV-infected PHH. RCN2 is an endoplasmic reticulum-localized calcium-binding protein that has been shown to influence apoptosis by regulating calcium levels within the endoplasmic reticulum [34,59]. We confirmed its downregulation by Western blot in PHH from two independent donors (Fig. 5), establishing RCN2 as a novel host protein affected during HBV infection. This regulation occurred at the post-transcriptional level.

Curiously, RCN2 is a known interaction partner of another oncogenic virus, human papillomavirus (HPV). It binds to the HPV E6 oncoprotein, although the functional significance of this interaction remains unclear [60]. Beyond viral infection, RCN2 has been linked to several cancers, including HCC, where its expression is typically increased in contrast to the reduction observed during HBV infection [61,62]. In this context, RCN2 has been reported to promote HCC cell proliferation, invasion and migration both *in vitro* and *in vivo* [61,62].

It remains unclear whether the reduction of RCN2 represents an active viral mechanism, analogous to HBx-mediated degradation of the Smc5/6 complex, or a secondary consequence of infection-induced stress. *In vitro*, siRNA-mediated knockdown of RCN2 did not alter markers of viral replication, indicating that its depletion does not affect HBV replication.

In the secretome, many proteomic changes were associated with BLV treatment; after excluding BLV-related effects, ten proteins remained differentially abundant in response to HBV infection (Fig. 3e). Applying a stricter filter that required consistent deregulation in both the HBV vs. HBV+BLV and HBV vs. Ctrl comparisons reduced this number to four proteins: APOB, XPNPEP3, ALDH4A1 and ENO3 (Table 2).

However, the increase in APOB secretion detected by MS could not be confirmed by ELISA, and APOB knockdown did not affect HBV replication in PHH (Figs 6 and S6). Interestingly, the association between APOB and HBV infection *in vitro* has been explored in previous studies. Qiao and Luo reported that silencing APOB did not affect HBV infection in HepG2-NTCP cells, which is consistent with our findings in PHH [39]. The impact of HBV on APOB expression has also been examined, with conflicting results. Norton *et al*. found no significant change in APOB mRNA levels in human hepatocytes stably transfected with the HBV genome [63], aligning with our observation that APOB transcript levels remained unchanged 8 dpi. In contrast, Wang *et al*. reported a reduction in both APOB mRNA and protein levels in HepG2 cells, following either stable or transient transfection with an HBV plasmid [64]. These discrepancies suggest that the regulation of APOB may depend on the experimental system, timing of analysis or the level of viral gene expression.

For the other secretome-associated proteins (XPNPEP3, ALDH4A1 and ENO3), Western blot analysis did not yield detectable signals, suggesting that their abundance in culture supernatants is below the detection threshold. To assess their potential role in HBV replication, we performed siRNA-mediated knockdown experiments in PHH. XPNPEP3 and ALDH4A1 were efficiently silenced at both the RNA and protein levels, but their depletion did not lead to consistent changes in viral replication markers (Fig. 8).

Nonetheless, the absence of an effect on replication does not rule out functional relevance. In particular, the proteins identified in the secretome may play roles beyond direct regulation of viral replication. For example, hepatocyte-derived extracellular vesicles are known to modulate immune responses and intercellular communication [65]. Moreover, while siRNA-mediated depletion is useful for identifying host dependency factors, it is less suited to uncover host restriction factors in HBV-infected PHH. This is because viral proteins can actively neutralize restriction pathways, preventing any observable increase in replication upon knockdown. A well-established example is the Smc5/6 complex, which is targeted for proteasomal degradation by HBx [32]. In this context, we cannot exclude the possibility that our siRNA screen may also have missed potential restriction functions of RCN2, which was likewise downregulated in HBV-infected PHH.

It is also possible that at least some of the observed proteomic alterations reflect a general cellular stress response rather than specific, functionally relevant host–virus interactions. The presence of intracellular proteins such as ALDH4A1, XPNPEP3 or ENO3 in the secretome can point to background from dying or leaky cells [66]. Alternatively, these proteins could be released as part of extracellular vesicles, and indeed, previous studies have reported their detection in extracellular vesicle proteomes [67,69]. However, the functional significance of their secretion remains unclear, and it is therefore uncertain why their abundance would change upon infection.

A limitation of our study is that some proteomic changes may not have been captured. The 8-day post-infection time point could have missed early or transient alterations, such as those linked to viral entry or cccDNA establishment. Additionally, technical constraints of MS-based proteomics also need to be considered. Our analysis identified more than 5,500 proteins, which is comparable to recent proteomic studies of PHH [20,70,72]. Nevertheless, certain proteins are likely absent due to methodological factors. Specific protein classes are systematically underrepresented in MS studies because of low abundance, poor ionization of hydrophobic peptides, limited tryptic cleavage sites or steric hindrance caused by extensive post-translational modifications. A typical example of such proteins are integral membrane proteins, which remain challenging to detect [73,74].

Taken together, our study underscores the importance of using carefully controlled experimental systems when analysing host responses to HBV infection *in vitro*. By combining an infection-based model with proteomic and secretomic profiling, and by accounting for inoculum- and drug-induced effects, we aimed to isolate changes specifically associated with productive HBV replication. We observed only a small number of differentially abundant proteins, consistent with the concept of HBV as a stealth virus that avoids triggering innate immune activation. RCN2 downregulation was confirmed in independent experiments, but functional assays based on siRNA-mediated knockdown of RCN2 and other candidates did not reveal a direct effect on HBV replication. The observed changes may therefore reflect general stress responses or, alternatively, contribute indirectly by modulating the hepatic microenvironment.

## Supplementary material

10.1099/jgv.0.002170 Uncited Supplementary Material 1.

10.1099/jgv.0.002170 Supplementary Material 2.

10.1099/jgv.0.002170 Uncited Supplementary Material 3.

10.1099/jgv.0.002170 Uncited Supplementary Material 4.

10.1099/jgv.0.002170 Uncited Supplementary Material 5.

## Abbreviations

ALDH4A1: delta-1-pyrroline-5-carboxylate dehydrogenase
APOB: apolipoprotein B
BLV: bulevirtide
CLIA: chemiluminescent immunoassay
CTG: CellTiter-Glo
ddPCR: digital droplet PCR
DIA: data-independent acquisition
dpi: days post-infection
ELISA: enzyme-linked immunosorbent assay
ENO3: beta-enolase
FC: fold change
FDR: false discovery rate
GSEA: gene set enrichment analysis
HBcAg: hepatitis B core antigen
HBeAg: hepatitis B e antigen
HBsAg: hepatitis B surface antigen
HBV: hepatitis B virus
HBx: regulatory protein X
HCC: hepatocellular carcinoma
HPV: human papillomavirus
IFA: immunofluorescence analysis
LC: liquid chromatography
LFQ: label-free quantification
MOI: multiplicity of infection
NSMCE3: non-structural maintenance of chromosomes element 3 homologue
NTCP: sodium taurocholate co-transporting polypeptide
PCA: principal component analysis
PEG: polyethylene glycol
PHH: primary human hepatocytes
RCN2: reticulocalbin-2
RT-qPCR: reverse transcription quantitative PCR
Smc5/6: structural maintenance of chromosomes 5/6
VGEs: viral genome equivalents
XPNPEP3: Xaa-Pro aminopeptidase 3
