# Supplementary material for: Hepatitis B virus is a stealth virus that minimizes proteomic and secretomic changes in primary human hepatocytes
Source: J Gen Virol. 2025 Nov 7;106(11):002170. doi: 10.1099/jgv.0.002170 (PMC12594339; doi:10.1099/jgv.0.002170)

Supplementary Material

Hepatitis B virus is a stealth virus that minimizes proteomic and secretomic changes in primary human hepatocytes

Karolína Štaflová[1], Kamila Clarová[1], Michal Doležal[1], Martin Hubálek[1], Alena Křenková[1], Jan Hodek[1], Iva Pichová[1]* and Aleš Zábranský[1]*

[1] Institute of Organic Chemistry and Biochemistry of the Czech Academy of Sciences, Prague, Czech Republic

*Correspondence

Aleš Zábranský, ales.zabransky@uochb.cas.cz; Iva Pichová, iva.pichova@uochb.cas.cz

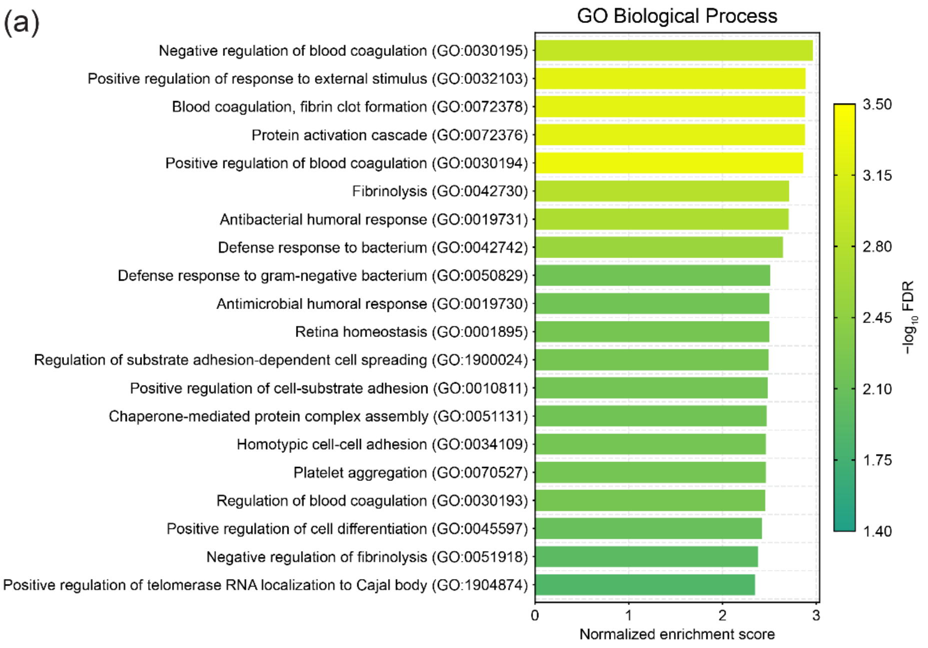

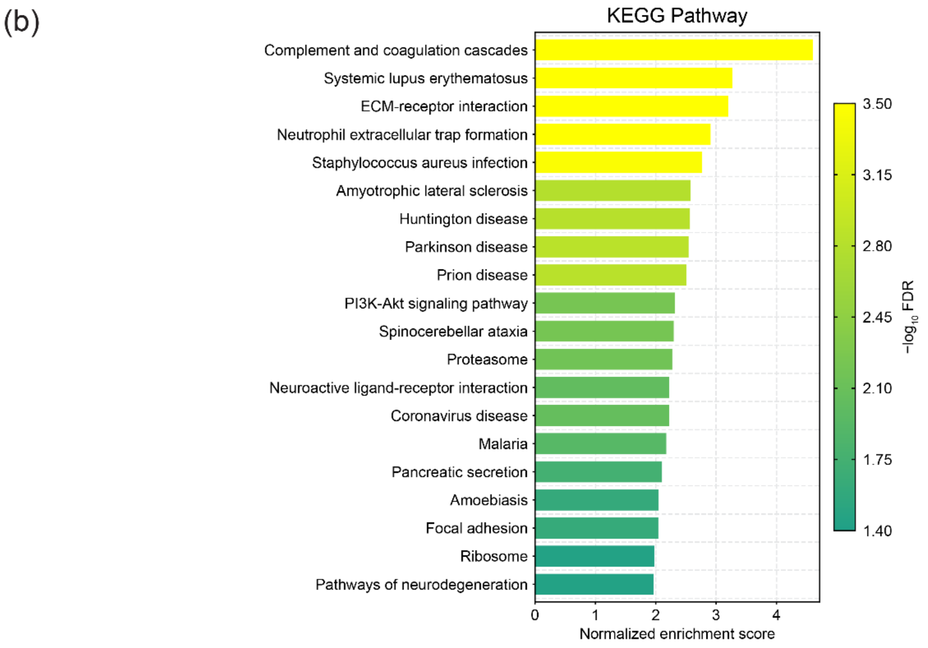

Fig. S1. Gene set enrichment analysis of proteins identified in the HBV inoculum. Proteins detected in the HBV inoculum were ranked by intensity and analyzed using GSEA. Shown are the top 20 enriched (a) Gene Ontology (GO) Biological Process terms and (b) KEGG pathways, ordered by normalized enrichment score. Statistical significance was assessed by permutation-based testing within the GSEA framework, and P values were adjusted for multiple testing using the Benjamini-Hochberg false discovery rate (FDR). The color scale indicates statistical significance as $-\log_{10}$(FDR).

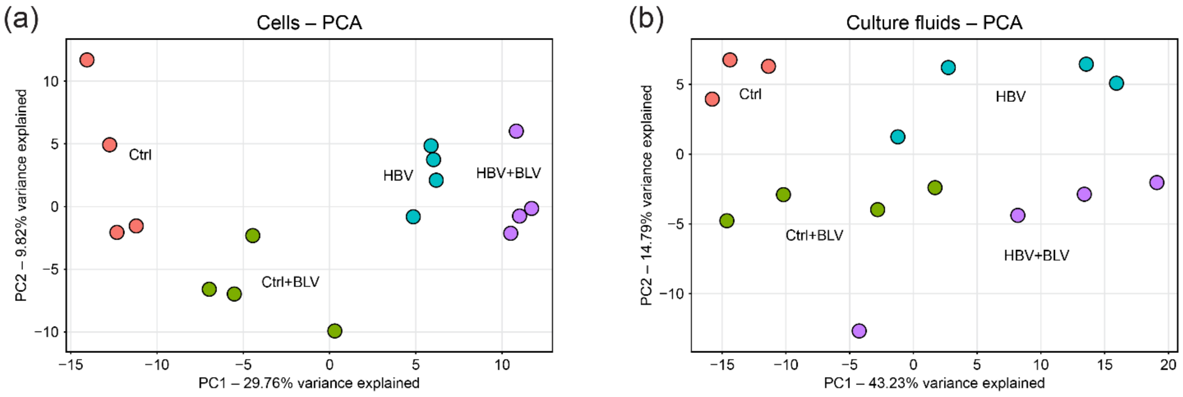

Fig. S2. Principal-component analysis (PCA) of proteomic data. PCA plots show the distribution of proteomic data for (a) cell lysates and (b) culture supernatants of primary human hepatocytes. Conditions include uninfected cells (Ctrl), BLV-treated cells (Ctrl+BLV), HBV-infected cells (HBV), and cells infected with HBV in the presence of BLV (HBV+BLV). Each point represents a biological replicate. Samples cluster according to experimental conditions, indicating condition-specific proteomic profiles.

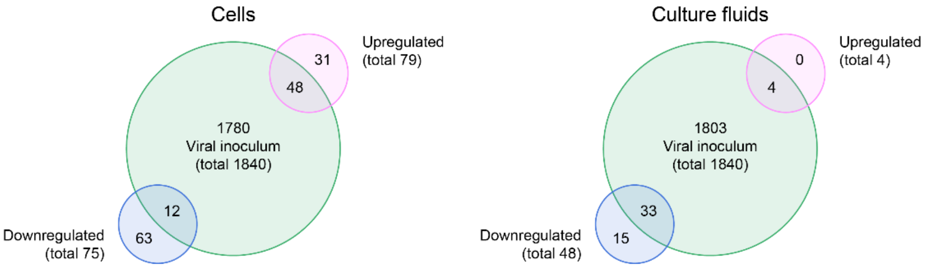

Fig. S3. Overlap between inoculum proteins and differentially abundant proteins in the HBV+BLV vs. Ctrl+BLV comparison. Venn diagrams show the overlap between proteins identified in the HBV inoculum and proteins differentially abundant at eight days post-infection in PHH lysates and culture fluids. Numbers indicate the number of proteins in each category. In supernatants, overlap was substantial, likely reflecting that both inoculum-derived and secreted proteins originate from the culture medium.

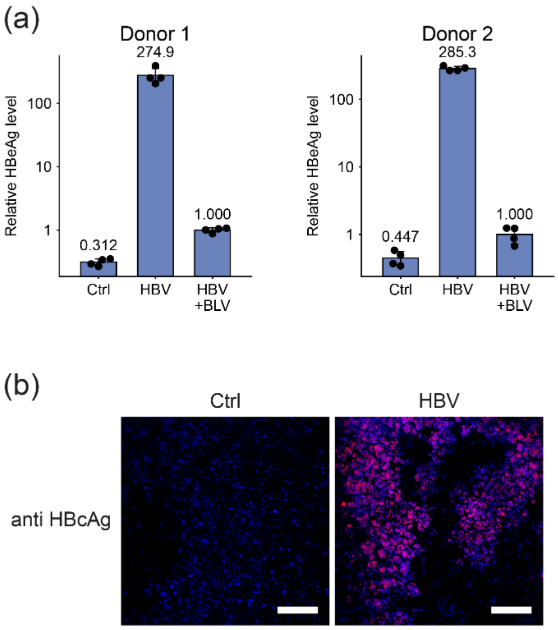

Fig. S4. Validation of HBV infection in PHH. PHH were infected with HBV at an MOI of 1000 VGE/cell, and infection was analyzed eight days post-infection. (a) Secreted HBeAg levels, determined by CLIA, are presented relative to HBV+BLV-treated cells on a logarithmic scale. (b) Infection efficiency in PHH from donor 2 was visualized by immunofluorescence staining for HBc, with approximately 40% of cells positive. Scale bar represents 300 μm.

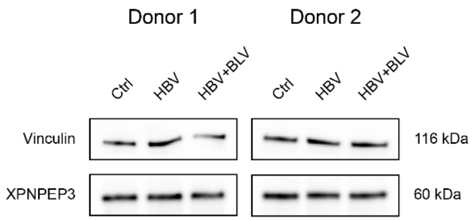

Fig. S5. Analysis of XPNPEP3 protein levels in HBV-infected PHH lysates. PHH were infected with HBV at an MOI of 1000 VGE/cell, and lysates were harvested eight days post-infection. XPNPEP3 protein levels were analyzed by Western blot.

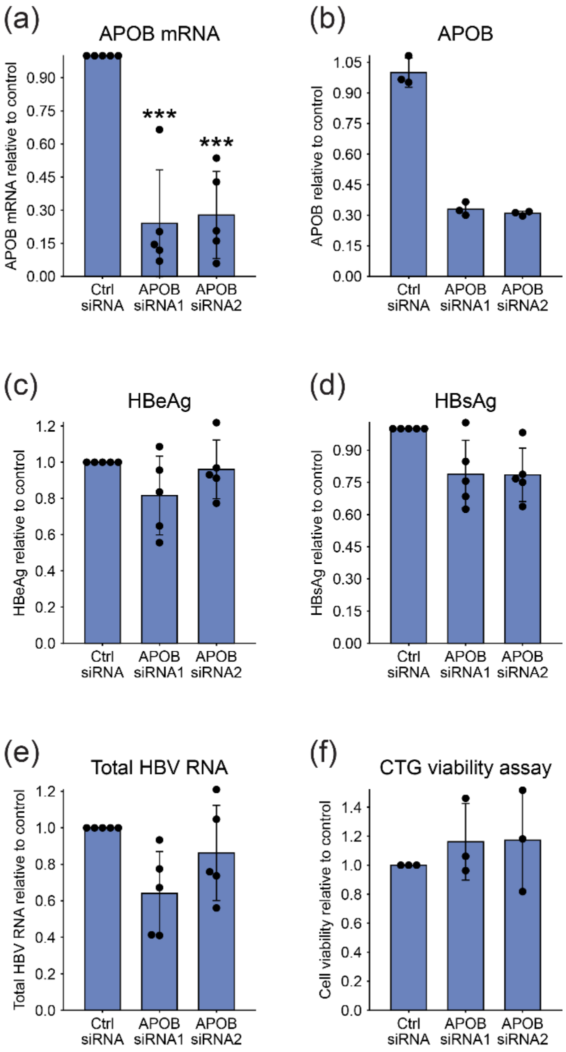

Fig. S6. Effect of APOB silencing on HBV infection in PHH. PHH were transfected with siRNAs targeting APOB and infected with HBV the following day at an MOI of 500 VGE/cell. Viral replication was assessed eight days post-infection. (a, b) Knockdown efficiency of APOB was assessed at the mRNA level by RT-qPCR (a) and at the protein level in culture supernatants by ELISA (b). (c, d) Levels of secreted HBeAg (c) and HBsAg (d) in culture supernatants were measured by CLIA. (e) Total intracellular HBV RNA was quantified by RT-qPCR. (f) Cell viability was assessed using the CellTiter-Glo assay nine days after siRNA transfection. Panels (a, c–e) represent mean ± SD from five independent experiments. Panel (f) represents three independent experiments. Each data point in panels (a, c-f) corresponds to the average of replicates within an individual experiment. Statistical significance between siRNA-treated groups and the control was determined by one-way ANOVA (*** $P < 0.001$). Panel (b) represents a single experiment performed in triplicate.

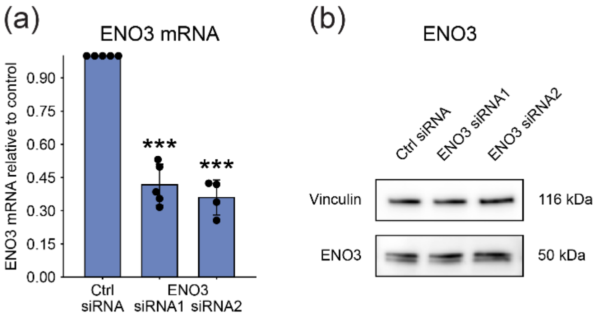

Fig. S7. Validation of ENO3 silencing in PHH. PHH were transfected with siRNAs targeting ENO3, infected with HBV the following day at an MOI of 500 VGE/cell, and analyzed nine days post-transfection. (a) ENO3 mRNA levels were quantified by RT-qPCR and are shown relative to control siRNA. Data represent mean ± SD from at least four independent experiments; individual points correspond to the average of technical replicates within each experiment. Statistical significance was determined by one-way ANOVA (***$P < 0.001$). (b) Western blot analysis of ENO3 protein levels in PHH lysates with vinculin as a loading control. Silencing was confirmed at the RNA level but not at the protein level.

Table S1 List of used primers

Primer	Primer sequence (5′ to 3′)
HBV DNA-F	GTGTCTGCGGCGTTTTATCA
HBV DNA-R	GACAAACGGGCAACATACCTT
HBV DNA-probe	FAM-TGAGGCATAGCAGCAGGATG-BHQ1
GAPDH-F	GAAGGTCGGAGTCAACGGATTT
GAPDH-R	CGTTCTCAGCCTTGACGGT
total HBV RNA-F	AGAGGACTCTTGGACTCTCAGC
total HBV RNA-R	CCTCCCAGTCTTTAAACAAACAGTC
ALDH4A1-F	CCATGGTGGGACAGGGTAAG
ALDH4A1-R	TCCACCGCATACTTGGCATT
APOB-F	ACCGGGGACACCAGATTAGA
APOB-R	AGGGTATCCACCAAGGCTCT
ENO3-F	TGACAGTCACCAACCCCAAG
ENO3-R	CCAGTTTGCACGCCTGGAT
RCN2-F	CAGGTGGGATCCAACTGCAA
RCN2-R	ACAGCTCTTGGGGATCAAGC
XPNPEP3-F	CTCCTCAGACCAGGGGAGGTA
XPNPEP3-R	GTTCTCGACTGGGATCTCGC

Table S2 List of used siRNAs

siRNA	Supplier	Sequence (5′ to 3′)
control siRNA	#4390843, Invitrogen	Not available
ALDH4A1 siRNA1	#s16483, Invitrogen	Sense: ACUUCUACAUCAACGACAA<br>Antisense: UUGUCGUUGAUGUAGAAGU
ALDH4A1 siRNA2	#s16484, Invitrogen	Sense: GAGUGGGACCUGAAGCCUA<br>Antisense: UAGGCUUCAGGUCCCACUC
APOB siRNA1	#s1476, Invitrogen	Sense: GCAAGUACCUGAGAACGGA<br>Antisense: UCCGUUCUCAGGUACUUGC
APOB siRNA2	#s1477, Invitrogen	Sense: CGUUUACCAUGACCAUCGA<br>Antisense: UCGAUGGUCAUGGUAAACG
ENO3 siRNA1	sc-37043, Santa Cruz Biotechnology	Not available
ENO3 siRNA2	#s223487, Invitrogen	Sense: AGUACGAUCUUGACUUCAA<br>Antisense: UUGAAGUCAAGAUCGUACU
RCN2 siRNA1	#12189, Invitrogen	Sense: GGAAGAUGUGGAUGAAUAU<br>Antisense: AUAUUCAUCCACAUCUUCC
RCN2 siRNA2	sc-62247, Santa Cruz Biotechnology	Not available
XPNPEP3 siRNA1	#121895, Invitrogen	Sense: GGUACUCCCUUCAGCCUGU<br>Antisense: ACAGGCUGAAGGGAGUACC
XPNPEP3 siRNA2	#s34286, Invitrogen	Sense: GCGCAGACAUUUUAGCCUA<br>Antisense: UAGGCUAAAAUGUCUGCGC

Uncropped western blot membranes

Left panels show the membranes with molecular weight markers (#A8889, AppliChem), right panels show the chemiluminescence detection. Red boxes indicate the areas cropped and shown in the main figure.

Figure 5: Donor 1; Vinculin

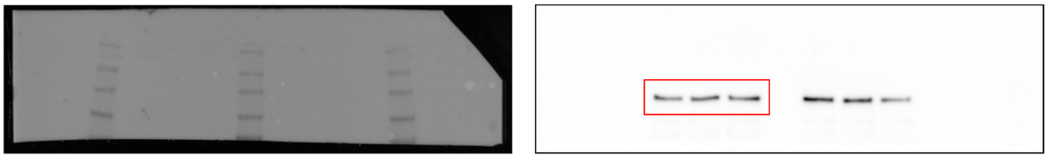

Figure 5: Donor 1; RCN2

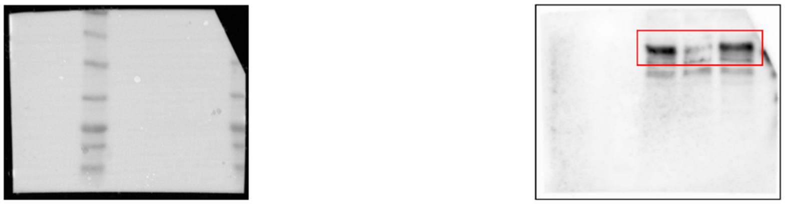

Figure 5: Donor 2; Vinculin

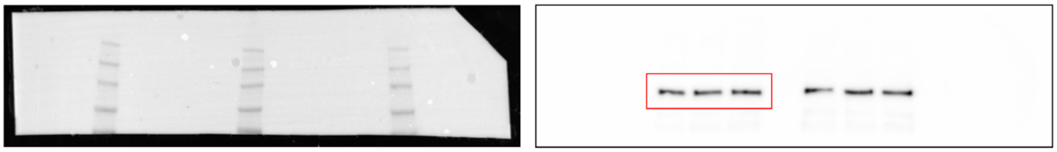

Figure 5: Donor 2; RCN2

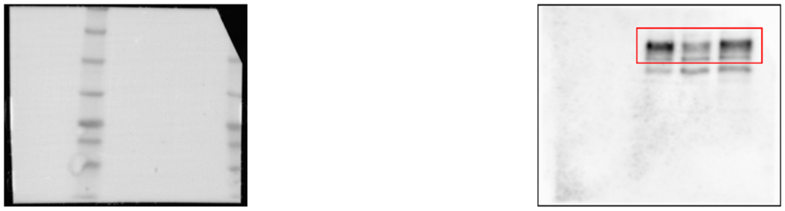

Figure 7b: Vinculin

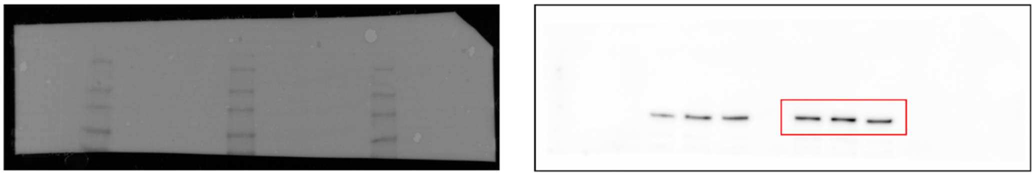

Figure 7b: RCN2

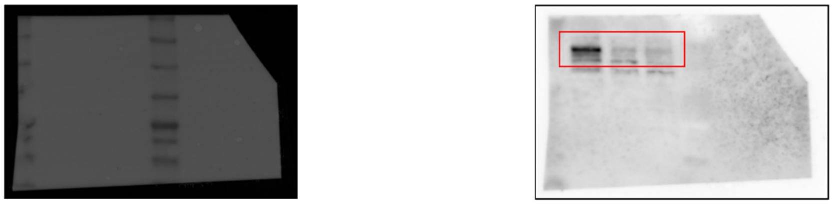

Figure 8a: Vinculin

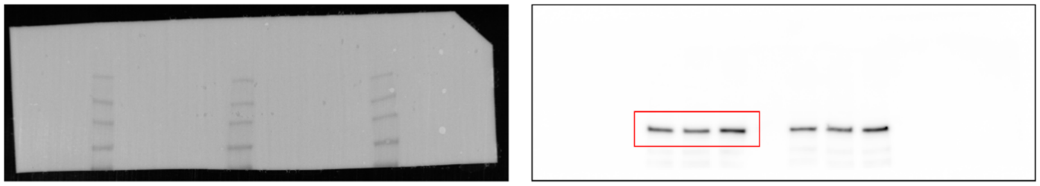

Figure 8a: XPNPEP3

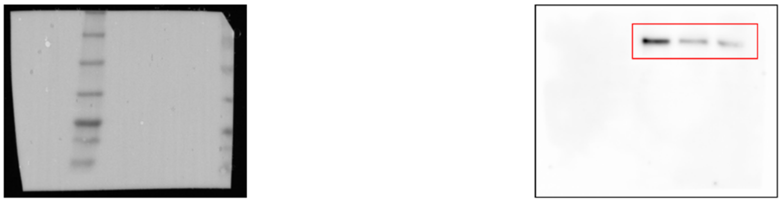

Figure 8b: Vinculin

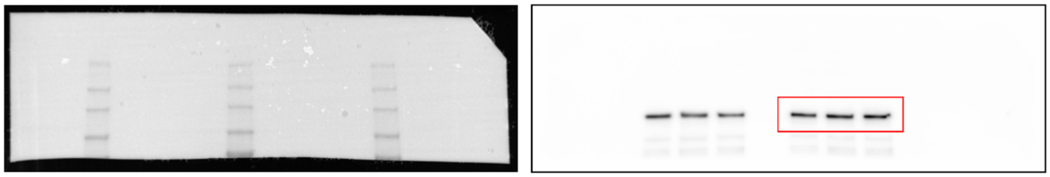

Figure 8b: ALDH4A1

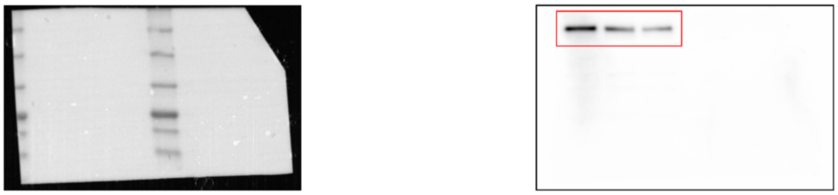

Figure S5: Vinculin

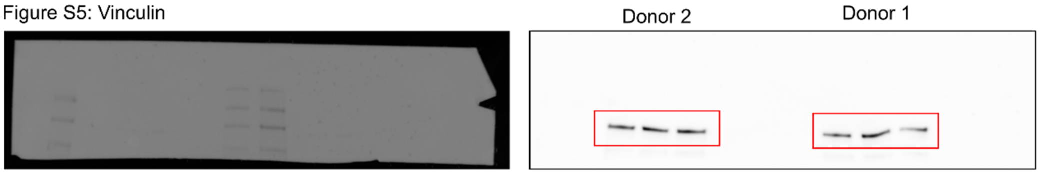

Figure S5: XPNPEP3

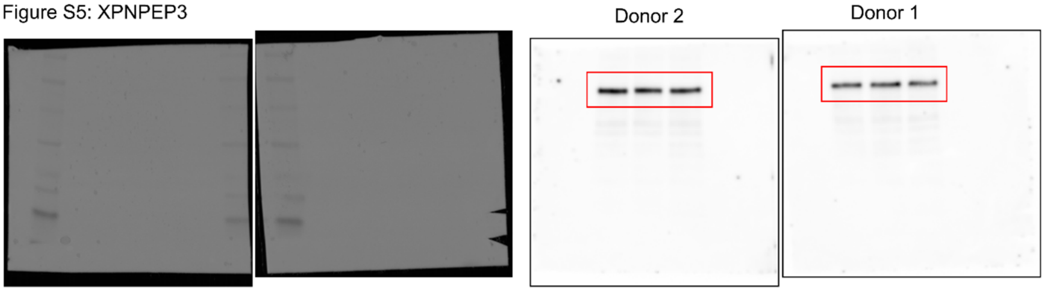

Figure S7: Vinculin

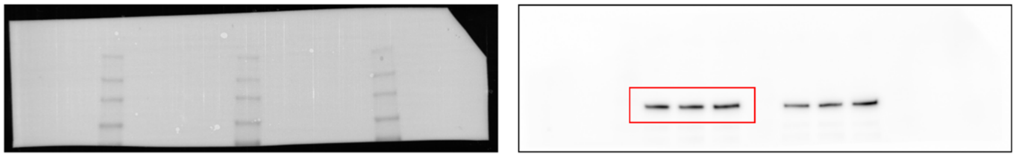

Figure S7: ENO3

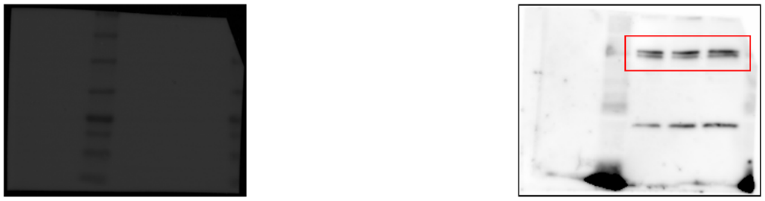

Supplement: Uncited Supplementary Material 1. [file jgv-106-02170-s001.pdf]
